# Addressing risk factors for hypertensive disorders in pregnancy: A case study of a quality improvement program in two states in Nigeria

**DOI:** 10.1371/journal.pone.0356879

**Published:** 2026-09-03

**Authors:** Ugo Okoli, Sylverius Obafemi, Comfort Okpe, Kendra Njoku, Nneka Mobisson, Chinyere Uzoama, Victoria Omoera, Emmanuel Ugwa, Kathleen Hill

**Affiliations:** 1 Jhpiego, an affiliate of Johns Hopkins University, Abuja, Nigeria; 2 mDoc Healthcare, Lagos, Nigeria; 3 Health & Human Services Secretariat, Abuja, Federal Capital Territory, Nigeria; 4 Ministry of Health, Lagos State Government, Lagos, Nigeria; 5 Department of Obstetrics & Gynaecology, Federal Medical Centre, Jigawa, Nigeria; 6 Jhpiego, an affiliate of Johns Hopkins University, Baltimore, Maryland, United States of America; Haramaya University, ETHIOPIA

## Abstract

Hypertensive disorders of pregnancy (HDP) including chronic hypertension, gestational hypertension preeclampsia and eclampsia are a leading cause of maternal and perinatal mortality in Nigeria and globally and increase the risk for future cardiovascular disease among women of reproductive age (WRA). The quality improvement program was implemented from 2019 through 2022 in the Federal Capital Territory (FCT) and Lagos State of Nigeria to improve early detection and management of HDP and risk factors (anemia, diabetes) at key maternal and reproductive healthcare touch points. A facility-based assessment evaluated provider knowledge and practices related to antenatal care diagnosis and management of HDP and risk factors in pregnant women in 20 facilities. From 2019 to 2022 the program implemented a package of interventions in 40 public and private health facilities to improve prevention, early detection, and management of HDP and risk factors among WRA during antenatal care (ANC), postnatal care (PNC), and family planning (FP) visits. ANC assessment results demonstrated important gaps in ANC providers’ knowledge, self-reported confidence and observed adherence with HDP and risk factor management best practices. Among 93,384 ANC visits across program facilities the median proportion of women who had a blood pressure, anemia and diabetes check increased from 44% to 89%, 48% to 91% and 32% to 87%, respectively, from baseline (October 2019-March 2020) to the program final 6 months (July-December 2022). From January 2021 to December 2022, the median proportion of 57,658 PNC visits with a documented BP check increased from 81% to 90% and the median proportion of 105,237 FP visits with a BP check increased from 73% to 98% from an initial baseline period (January- June 2021) to the last 6 months of program implementation (July-December 2022). In a program setting with a high burden of maternal mortality and morbidity due to hypertensive disorders and a high prevalence of cardiovascular disease, improving identification and management of HDP and risk factors at key health care touch points for women of reproductive age has the potential to reduce HDP morbidity and mortality and future premature cardiovascular mortality among women of reproductive age.

## Introduction

Hypertensive disorders of pregnancy (HDP) including preeclampsia, eclampsia, gestational hypertension, and chronic hypertension are leading causes of maternal mortality, preterm birth, and stillbirth in Nigeria and globally [1,2]. Globally, hypertensive disorders are the third most common cause of maternal death (16%) after hemorrhage (27%) and indirect obstetric deaths (23%) [3].The United Nations Maternal Mortality Estimation Inter-Agency Group (MMEIG) estimated the Nigeria maternal mortality ratio (MMR) to be 993 deaths per 100,000 live births in 2023 [4], modestly decreased from an estimated 1,065 per 100,000 live births in 2020.In a cross-sectional analysis of maternal and perinatal data across 54 tertiary-level facilities in Nigeria, hypertensive disorders were the leading cause of maternal deaths accounting for 31.8% of maternal deaths [5]. Approximately 5–10% of pregnancies in Nigeria are complicated by hypertensive disorders [6]. Globally, the prevalence of HDP is 116.4 per 100,000 women of childbearing age; sub-Saharan Africa has the highest prevalence of HDP, with a mean prevalence of 334.9 per 100,000 women of childbearing age [7]. A secondary analysis of global and regional trends in data from the Global Burden of Disease 2021 study, estimated that western Sub-Saharan Africa has the highest age-standardized incidence rate of HDP among women aged 15–49 years in the world at 3,378.94 per 100,000 females and that central sub-Saharan Africa has the highest age-standardized HDP mortality rates in the world at 8.27 per 100,000 population [8]. A systematic review and meta-analysis of prevalence and materno-fetal outcomes of pre-eclampsia and eclampsia (PE/E) among pregnant women in Nigeria revealed a pooled prevalence of 4.51% for preeclampsia and 1.39% (95% CI 1.02–1.84) for eclampsia and a pooled maternal mortality rate associated with PE/E of 6.04% (95% CI 3.67–8.89) and pooled fetal mortality rate of 16.73% [9].

In addition to being a leading cause of maternal deaths, hypertensive disorders of pregnancy increase a woman’s risk for future cardiovascular disease and premature mortality and morbidity due to stroke, myocardial infarction, and heart failure and increase risk for adverse perinatal outcomes including but not limited to stillbirth, preterm birth, and low birth weight [10,11]. Importantly, the burden of premature deaths from HDP and associated cardiovascular disease (CVD) later in life falls disproportionately upon LMICs [12].

Globally, non-communicable diseases (NCDs) including hypertension (HTN), cardiovascular disease, and diabetes are the leading causes of death and disability in all women, including in women of reproductive age [2]. Anemia and diabetes increase risk for HDP, including pre-eclampsia and eclampsia, and women with gestational diabetes are at higher risk for both gestational hypertension and preeclampsia [13,14]. In a secondary analysis of a facility-based 2004–2005 WHO Global Survey on Maternal and Perinatal Health history, chronic hypertension and severe anemia increased the risk of pre-eclampsia/eclampsia by three times or more [15]. HDP risk factors are additive; the presence of more than one HDP risk factor, such as anemia and diabetes, increases the risk of HDP and linked adverse maternal and perinatal outcomes (e.g., preterm birth; low birth weight) [16].

The prevalence of hypertension in Nigeria (among all populations) is estimated at 31%, with low patient awareness (29%), treatment (12%), and control (2.8%) rates [17]. Public awareness of the burden of NCDs and the need for primary health care across the life course is generally low. Quality and utilization of primary health care services is low among women of reproductive age, and pregnant women are often excluded from PHC, NCD and HTN programs and studies [18]. A 2019 community-based assessment of women of reproductive age (WRA) in the four program LGAs and ACs measured a high burden of hypertension and pre-hypertension (36%) (based on Nigeria national guidelines), moderate or severe anemia (19%), and pre-diabetes or diabetes (9.6%) among 400 WRA [19,20] and low knowledge of HDP risk factors among 649 surveyed WRA [19,20].

Maternal and reproductive health services offer an important and underutilized platform in Nigeria to reach WRA at key health care touchpoints to prevent, detect and manage HDP and risk factors to reduce maternal and perinatal mortality and future risk for premature cardiovascular disease mortality – a triple win. Approximately 67% of pregnant women in Nigeria access at least one antenatal care visit [21]. However, systematic assessment of HDP and risk factors as part of maternal and reproductive health care services is often lacking contributing to important missed opportunities to identify and mitigate risk in women with HDP and risk factors. A study of HTN treatment and control among pregnant women in 60 primary health centers in an HTN treatment program in Federal Capital Territory (FCT) showed that pregnant women were less likely to be treated for HTN than their non-pregnant counterparts and demonstrated undertreatment and inappropriate treatment of HTN and poor control of blood pressure in pregnant women in program sites and no use of aspirin for primary prevention in pregnant women with HTN [22]. Improving detection and management of HDP and risk factors at key maternal and reproductive health care touch points has the potential to reduce maternal and perinatal mortality and morbidity associated with HDP and to increase primary and secondary strategies for prevention of cardiovascular disease in women of reproductive age.

The Reducing Indirect Causes of Maternal Mortality and Morbidity (RICOM3) program was implemented from 2019 to 2022 in 40 public and private primary and secondary healthcare facilities in the Federal Capital Territory and Lagos State to improve early detection and management of HDP and modifiable risk factors (HTN, anemia, diabetes) at key maternal and reproductive health care touch points including routine family planning, antenatal and postnatal care visits.

The program leveraged the Federal Ministry of Health National Multisectoral Action Plan for Prevention and Control of Non-communicable diseases (2019–2025) and Nigeria’s membership in the multi-country Network for improving quality of care for maternal, newborn and child health [23].

## Methods

Program sites: The Reducing Indirect Causes of Maternal Mortality and Morbidity (RICOM3) program was implemented in one urban and one peri-urban Local Government Authority (LGA) in Lagos State (Alimosho and Ikorodu) and one urban and peri-urban Area Council (AC) in the Federal Capital Territory (AMAC and Bwari), beginning in a first wave of 20 healthcare facilities from 2019–2020 (10 facilities in Lagos state and 10 facilities in FCT) and extending to an additional 20 facilities for a total of 40 healthcare facilities from 2021–2022 (20 facilities in Lagos State and 20 facilities in FCT). Program facilities included 8 public general (secondary) hospitals, 10 private hospitals and 22 primary health centers across the two states. Facilities were selected in collaboration with the government to include a mix of public and private primary and secondary sites providing a minimum volume of maternal services (at least 20 births per month).

Facility Assessment of ANC Services (2019): An assessment of ANC services was conducted in 2019 in 20 program facilities (6 public general hospitals, 4 private hospitals, and 10 PHCs) to inform the design of the program interventions. Assessment methods included observation of 40 ANC visits to evaluate screening for HDP and risk factors (anemia, diabetes) and a provider written questionnaire to assess knowledge and self-reported confidence for prevention, diagnosis and management of HDP and risk factors.

### Program interventions (2019–2022)

Program interventions and activities were designed in collaboration with local government and stakeholders drawing on existing local assets and priorities and findings from the initial facility-based ANC assessment and a 2019 community-based assessment conducted by the program in program areas and published separately [19,20].

**Fig 1:**
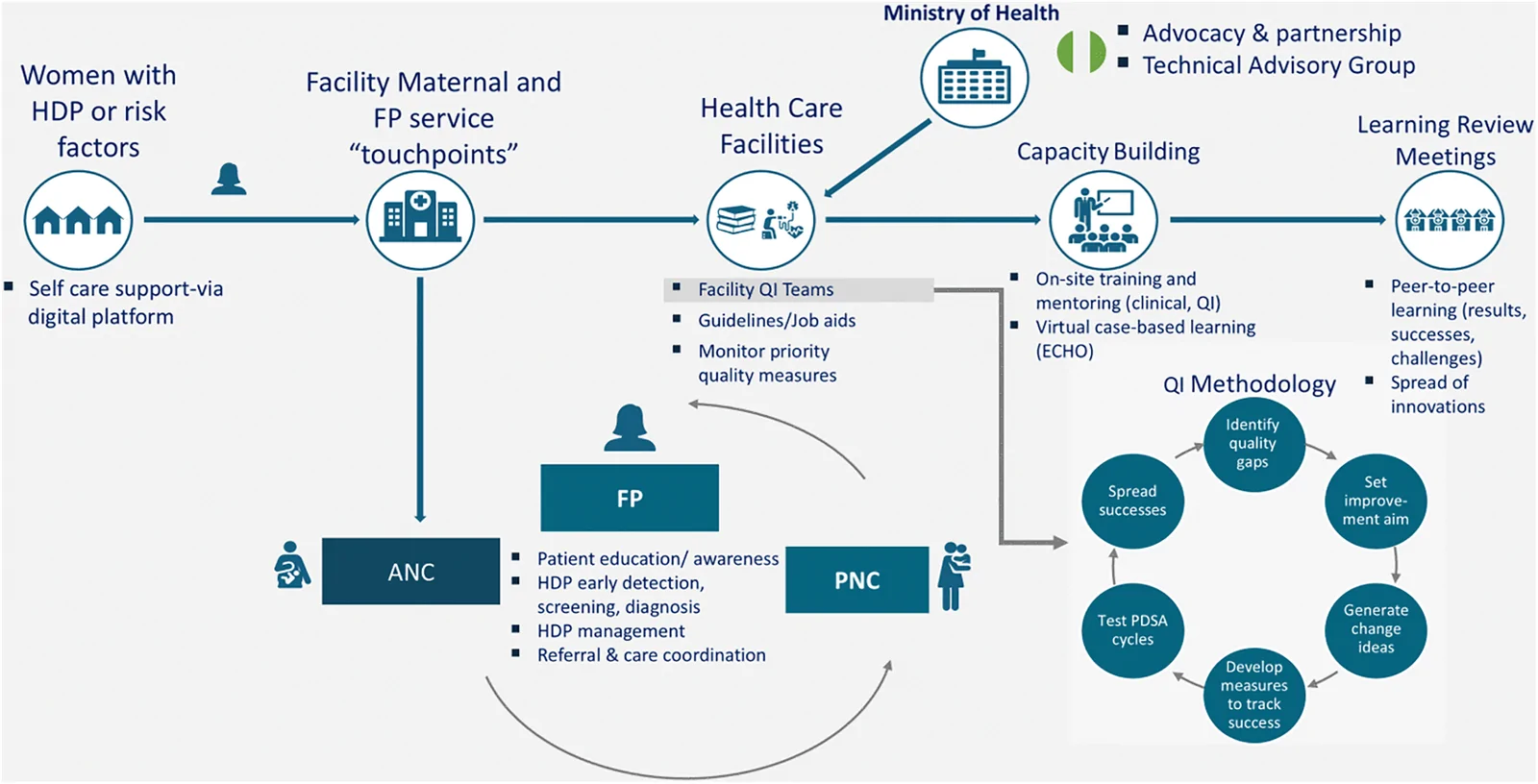
Program interventions to improve prevention, screening and management, including self-care, of HDP and risk factors among women using ANC, PNC and FP services in program facilities.

Program interventions included four broad categories of interventions implemented simultaneously in 20 facilities from October 2019 through December 2020 and in 40 facilities from 2021–2022 (Fig 1): 1) on-site competency-based training followed by blended on-site and virtual mentoring to strengthen provider knowledge and clinical skills for screening and management of HDP and risk factors as part of routine ANC, postnatal care (PNC) and FP services. Initial training was complemented by bi-monthly virtual case-based learning sessions led by local experts to strengthen clinical problem solving skills (applying the ECHO model); 2) monthly mentoring (on-site or virtual) for formation and optimal functioning of facility QI teams; mentoring focused on building skills for analyzing root causes of quality gaps in assessment and management of HDP and risk factors, implementing iterative QI cycles to improve routine screening and management of HDP and risk factors (anemia, diabetes) during facility ANC visits (2019–2022) and during PNC and FP visits (2021–2022) and regularly calculating, plotting and analyzing trends in quality of care indicators; 3) bi-annual cross-facility learning review meetings to share results and learning across sites including local solutions for improving detection and management of HDP and risk factors during ANC, PNC and FP visits; 4) rolling enrollment of women of reproductive age of women with HDP and risk factors onto a digital health platform for ongoing self-care support. In addition, the program supported modest distribution of blood pressure cuffs and glucometers in selected facilities. The program supported the formation of a Technical Advisory Group in each state to help guide the design of the program interventions, to provide periodic feedback on program interventions and results and to help advocate for increased resources, favorable policies, and engagement of key stakeholders, including State and LGA health managers, in longer-term efforts to improve identification and management of HDP and risk factors associated with preventable maternal mortality and morbidity and premature cardiovascular disease as part of maternal, reproductive and primary health care services.

### Program monitoring including data collection and analysis during implementation of project interventions (2019–2022)

In collaboration with facility managers, the program selected 5 quality-of-care indicators to monitor adherence with screening of HDP and risk factors in women attending ANC, PNC and FP visits in program facilities. Three ANC quality of care indicators were monitored in the first wave of 20 program facilities from October 2019 to December 2020 and in a subsequent expanded wave of 40 program facilities from January 2021- December 2022 (% pregnant women screened for HTN during ANC visits; % women screened for diabetes during ANC visits; % women screened for anemia during ANC visits). Two QoC indicators were introduced when the program expanded to 40 sites and initiated activities to improve HDP and risk factor screening during PNC and FP services (% women screened for HTN during PNC visits; % women screened for HTN during FP visits).

Facility QI teams were trained and supported to calculate QoC indicators using data extracted from existing facility ANC, PNC and FP registers on a monthly basis and were mentored to plot indicator results in a run chart (e.g., Fig 6) and apply the run chart shift rule to interpret results. A baseline and endline (July – December 2022) median were calculated based on the 6 initial data points and final program 6 data points for each indicator respectively. QI teams were supported to analyze trends in the data to guide iterative rapid QI cycles to improve adherence with screening for HDP and risk factors as part of routine ANC, PNC and FP visit workflows. Health care workers’ (HCWs) knowledge was assessed with a standard questionnaire before and after on-site clinical training in 20 program sites in 2021.

## Results

Results are presented for the 2019 facility ANC assessment and for indicators monitored during program implementation in supported facilities in Lagos State and the FCT.

### Facility Assessment of ANC Services (2019)

#### Healthcare worker knowledge and confidence in diagnosing and managing HDP and risk factors.

**Fig 2:**
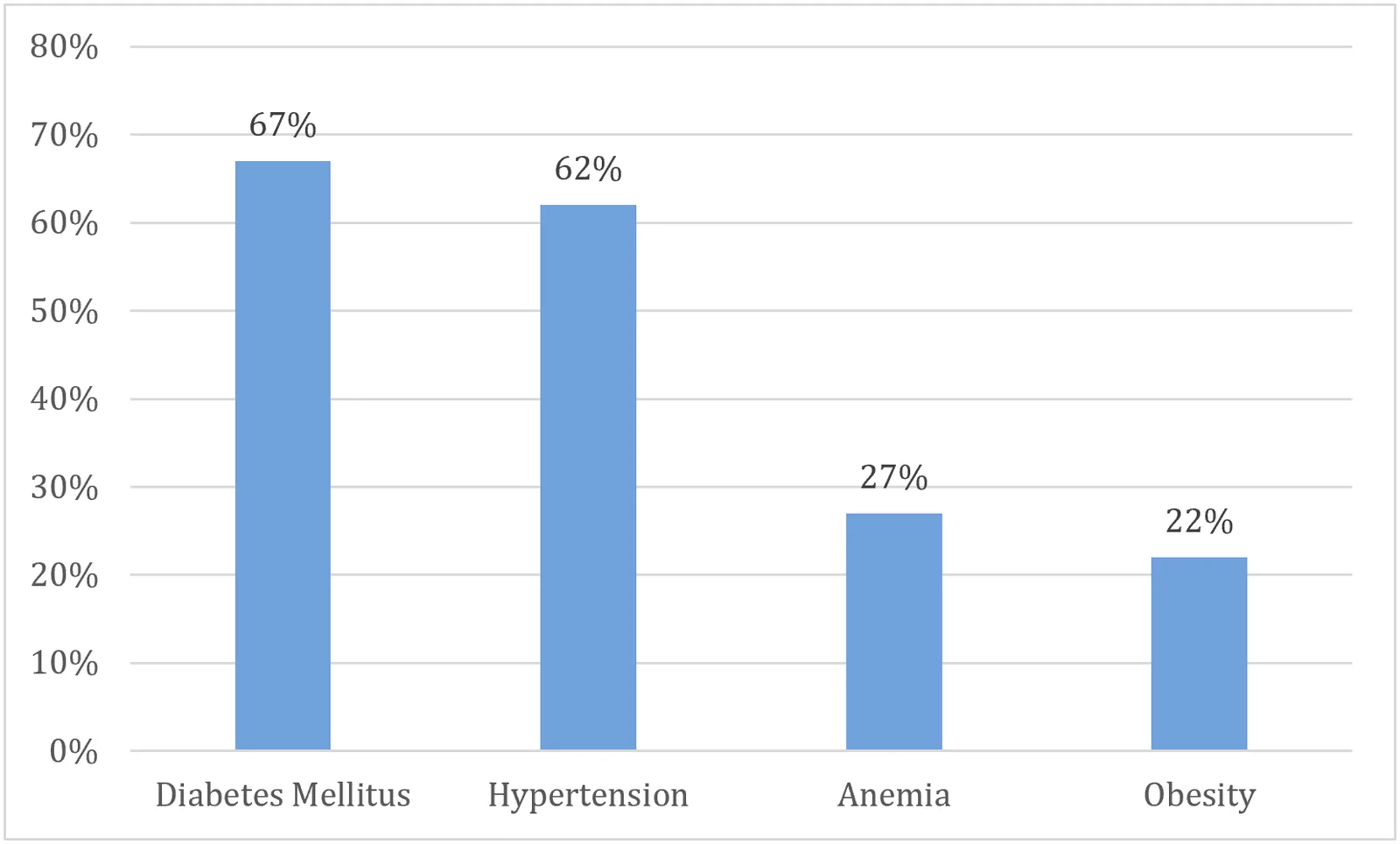
Percentage of health care workers who correctly identify diagnostic criteria for diabetes mellitus, HTN, anemia and obesity (n = 79 surveyed HCWs in 20 program sites).

Seventy-nine providers of ANC services across the 20 first-wave program facilities completed a questionnaire administered during on-site assessment visits, including 57 (72%) nurse/midwives, 10 (13%) midwives, 7 (9%) community health extension workers, and 5 (6%) community health officers. Two thirds (62%) of HCWs correctly identified diagnostic criteria for diabetes and HTN respectively and 27% and 22% of HCWs correctly identified diagnostic criteria for anemia and obesity (Fig 2). By contrast, self-reported confidence in diagnosing hypertension and PE/E was higher among 79 surveyed HCWs at 84% and 71% respectively (Table 1). Provider self-reported confidence in treating hypertension and PE/E was lower at 62% and 49% respectively.

**Table 1:** Percentage of HCWs expressing confidence to diagnose and treat HTN, PE/E, Diabetes Mellitus (DM) & Anemia in pregnant women (n = 79 surveyed HCWs in 20 program sites).

% HCWs who expressed confidence to diagnose and treat HTN, PE/E, Diabetes Mellitus, Anemia and to interpret BMI values in pregnant women			
	Lagos	FCT	Average
Diagnose HTN	77%	91%	84%
Treat HTN	67%	57%	62%
Diagnose PE/E	67%	74%	71%
Treat PE/E	62%	36%	49%
Diagnose Diabetes	62%	46%	54%
Treat Diabetes	42%	23%	33%
Diagnose Anemia	55%	64%	60%
Treat Anemia	62%	54%	58%
Interpret BMI values	47%	23%	35%

*HTN – Hypertension; PE/E – Pre-eclampsia and eclampsia; BMI – Body mass index.

Two thirds (67%) and one quarter (27%) of surveyed HCWs correctly identified diagnostic criteria for diabetes and anemia respectively, while 54% and 60% of HCWs self-reported confidence in diagnosing diabetes and anemia respectively. One third (38%) and 58% percent of HCWs reported confidence in treating diabetes and anemia respectively. Only 35% of HCWs reported confidence in interpreting body mass index (BMI) values.

**Fig 3:**
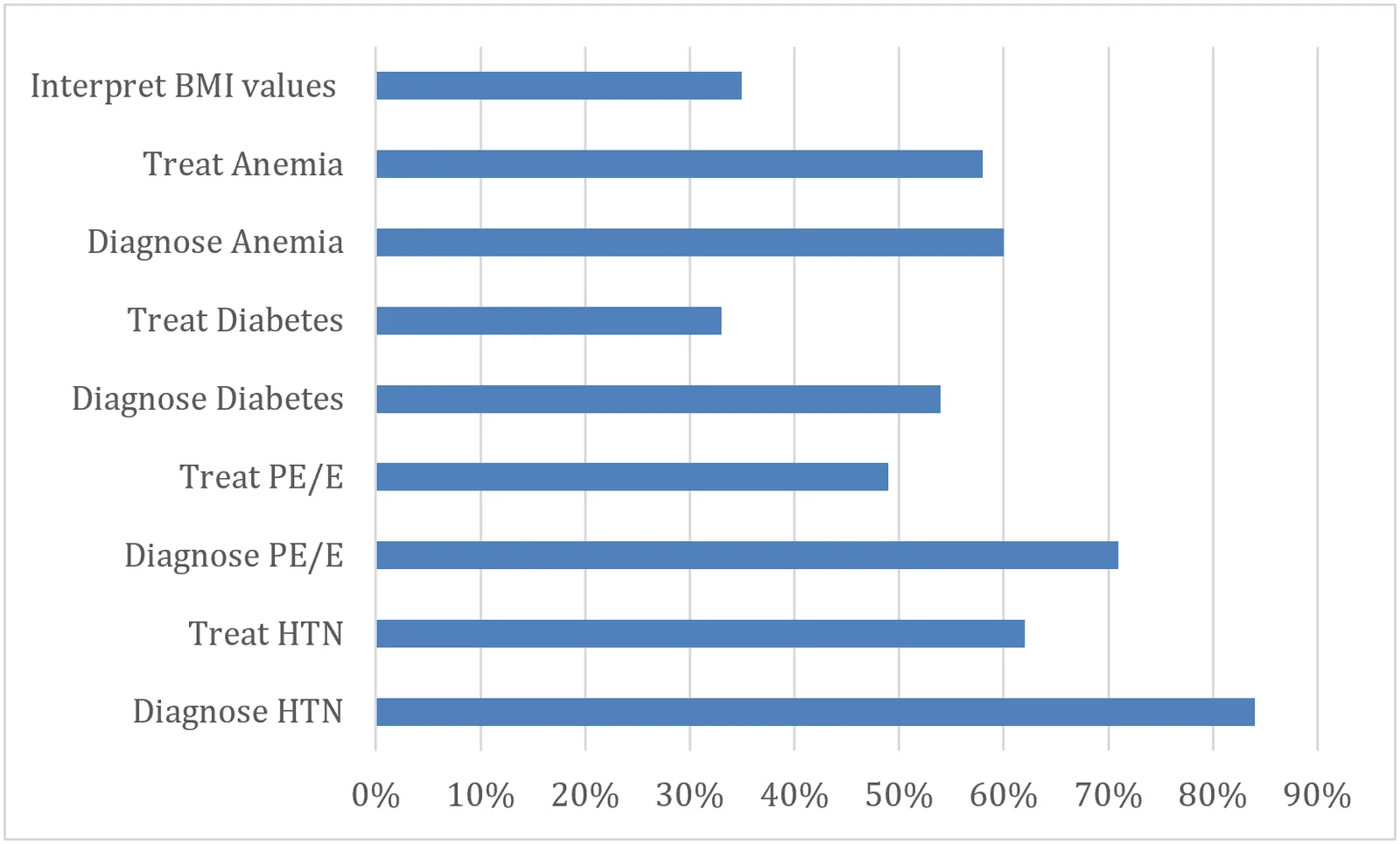
Percent of observed ANC visits demonstrating best practice for identification of HDP risk factors in pregnant women (N = 40 observed ANC visits in 20 program healthcare facilities in FCT and in Lagos state).

Adherence to best practices for identification of HDP and risk factors during ANC visits Observation of 40 ANC visits in 20 program facilities (Fig 3) demonstrated variable provider adherence to best practices for identifying HDP and risk factors (anemia, diabetes) in pregnant women. Providers asked about a family history of hypertension and Diabetes in 65% and 70% of visits respectively and asked about a personal history of pre-eclampsia/eclampsia in only 38% and 43% of visits respectively. Only one quarter of providers assessed women for appropriate blood pressure cuff size prior to measurement of blood pressure.

### Results During Program Implementation (2019–2022)

#### Healthcare worker knowledge gains following training.

**Fig 4:**
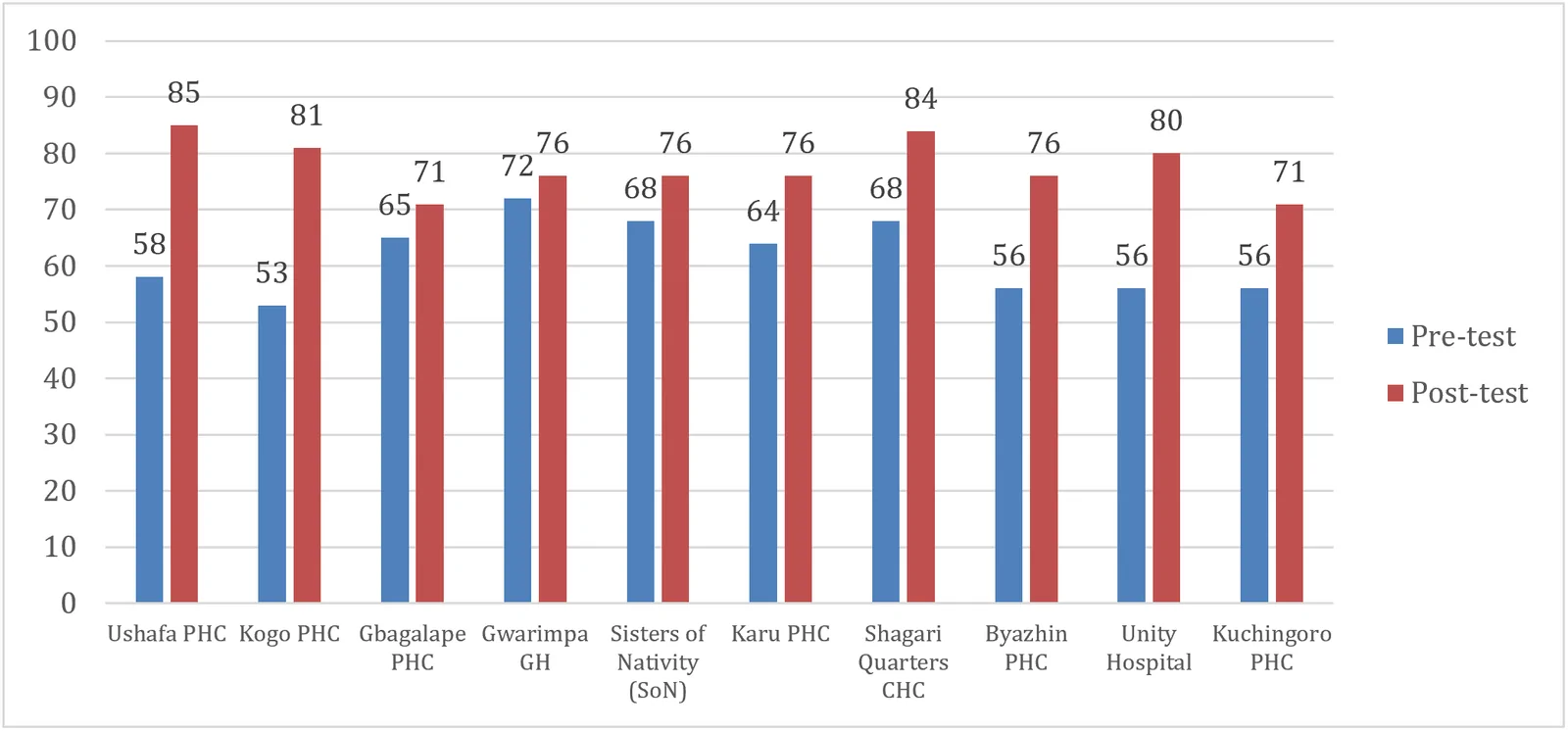
Pre and Post Training Knowledge of HCWs in FCT (n = 152 HCWs completing written knowledge assessment 2021).

**Fig 5:**
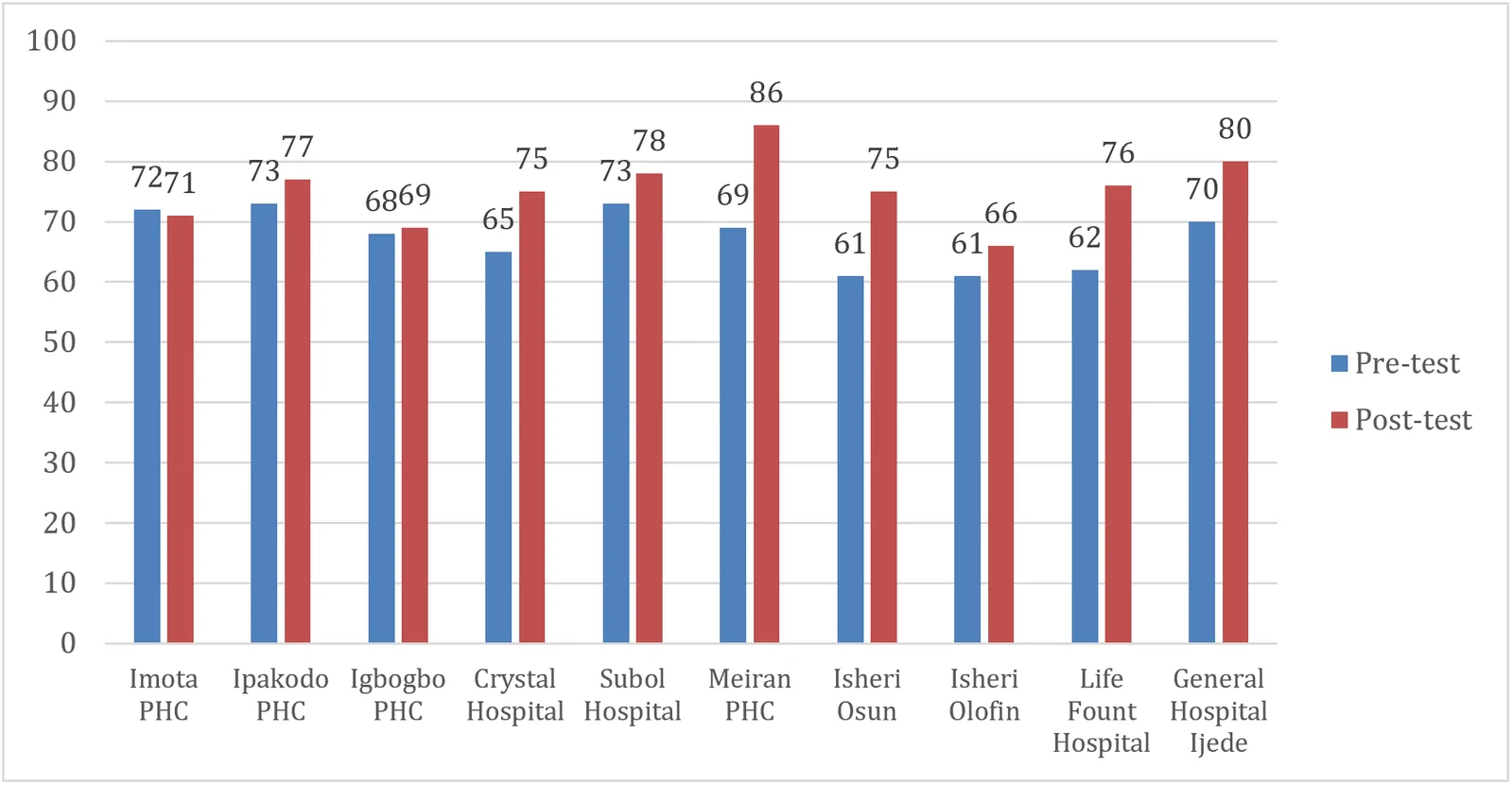
Pre and Post Training Knowledge of HCWs in Lagos State (n = 136 HCWs completing knowledge assessment 2021).

288 HCWs across 20 facilities completed pre- and post-training knowledge assessments in 2021 (Figs 4 and 5). Average knowledge scores increased by 12% overall, with greater gains observed among HCWs in the Federal Capital Territory (16.1%) compared to Lagos State (8.1%). Post-training, 90% of HCWs achieved a knowledge score of at least 80%, indicating improved capacity to identify and manage HDP and risk factors (anemia, diabetes).

#### Documentation of blood pressure, diabetes and anemia checks during ANC visits (20 facilities October 2019-December 2020; 40 facilities January 2021-December 2022).

**Fig 6:**
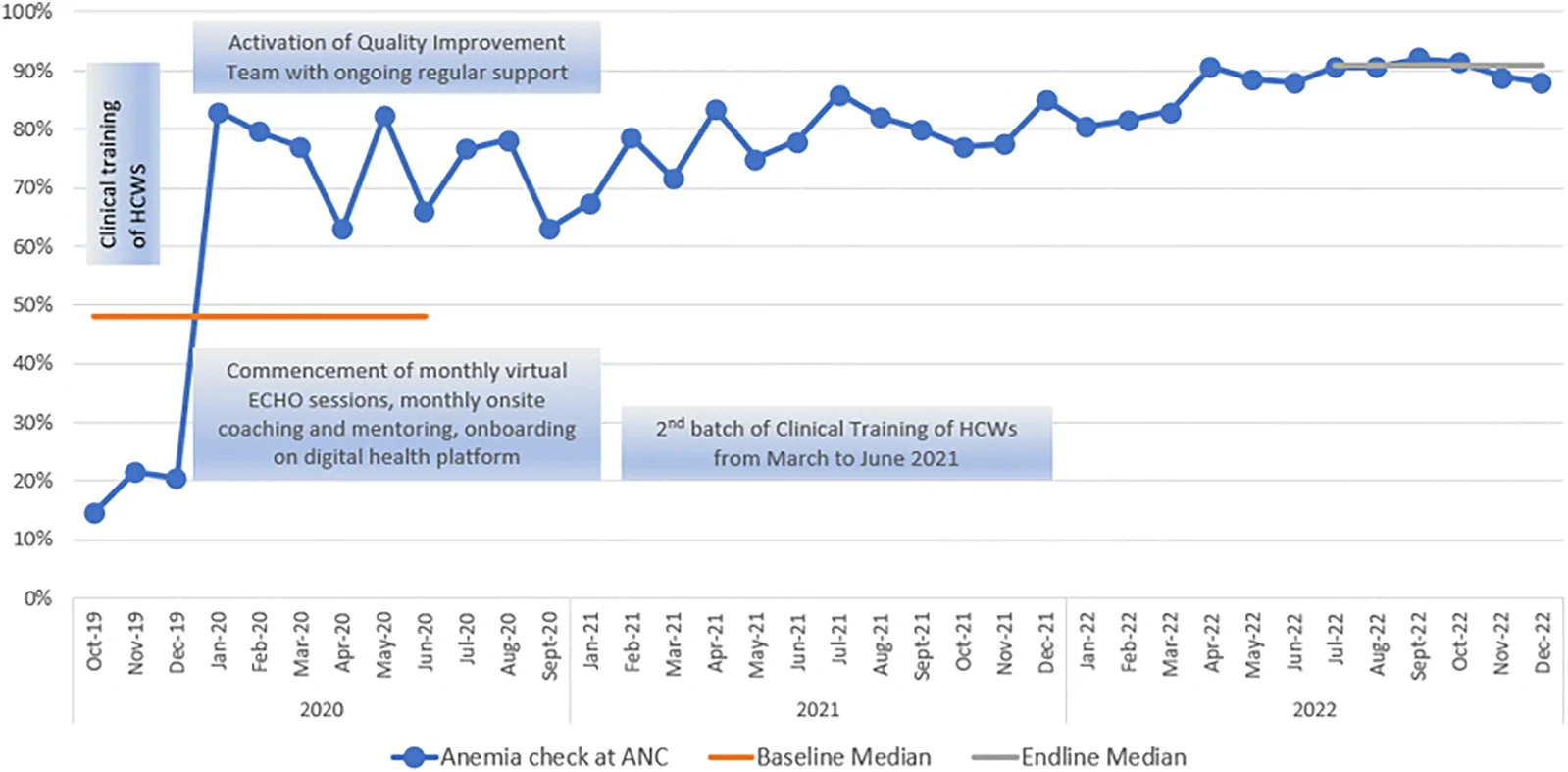
Percentage of ANC visits with hemoglobin test documented to assess for anemia in Lagos & FCT (n = 93,384 ANC visits in 20 facilities 2019-2020 and 40 health facilities 2021 - 2022).

**Fig 7:**
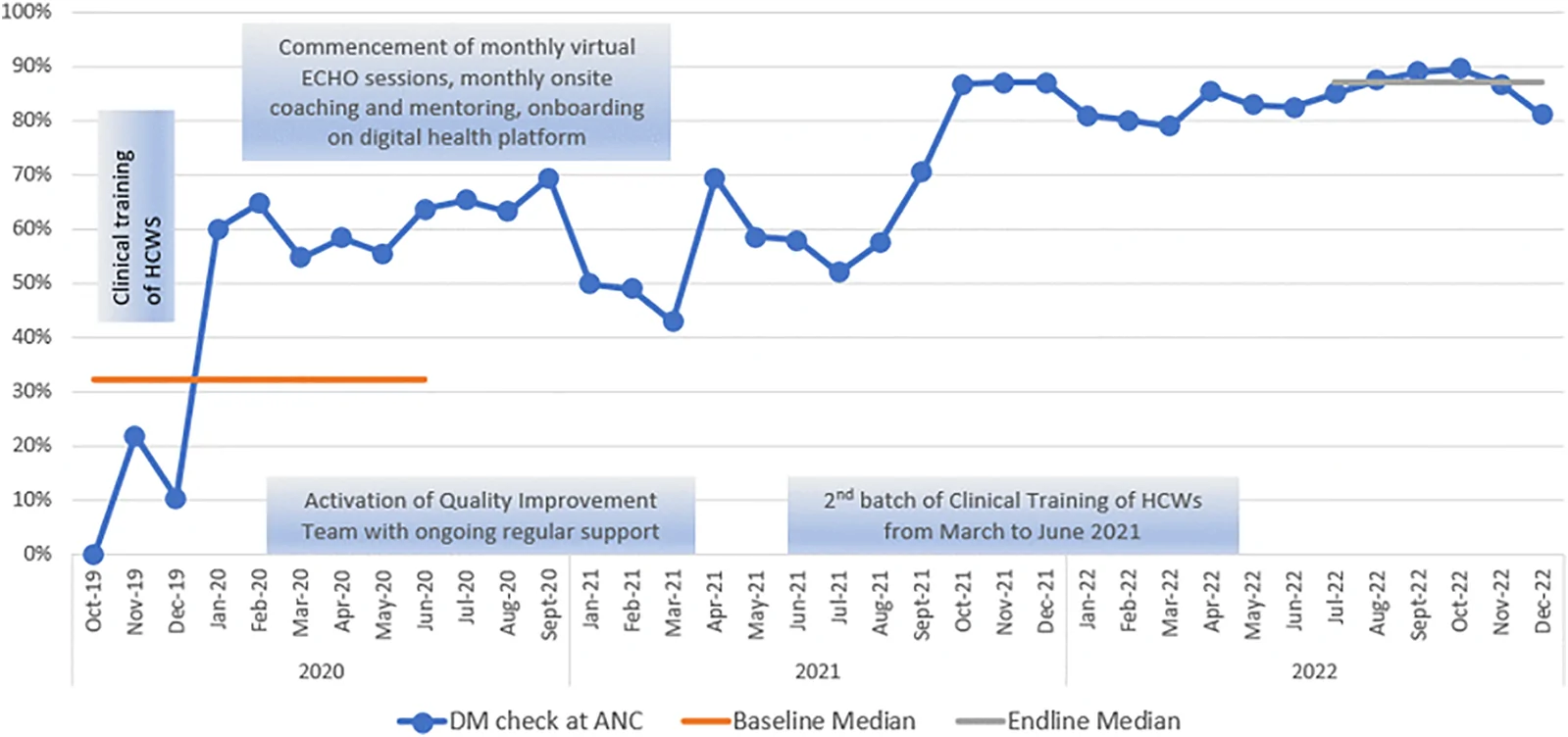
Percentage of ANC visits with glucose test documented to assess for diabetes in Lagos and FCT (n = 93,384 pregnant women attending ANC in 20 facilities in 2019-2020 and 40 health facilities 2021-2022).

**Fig 8:**
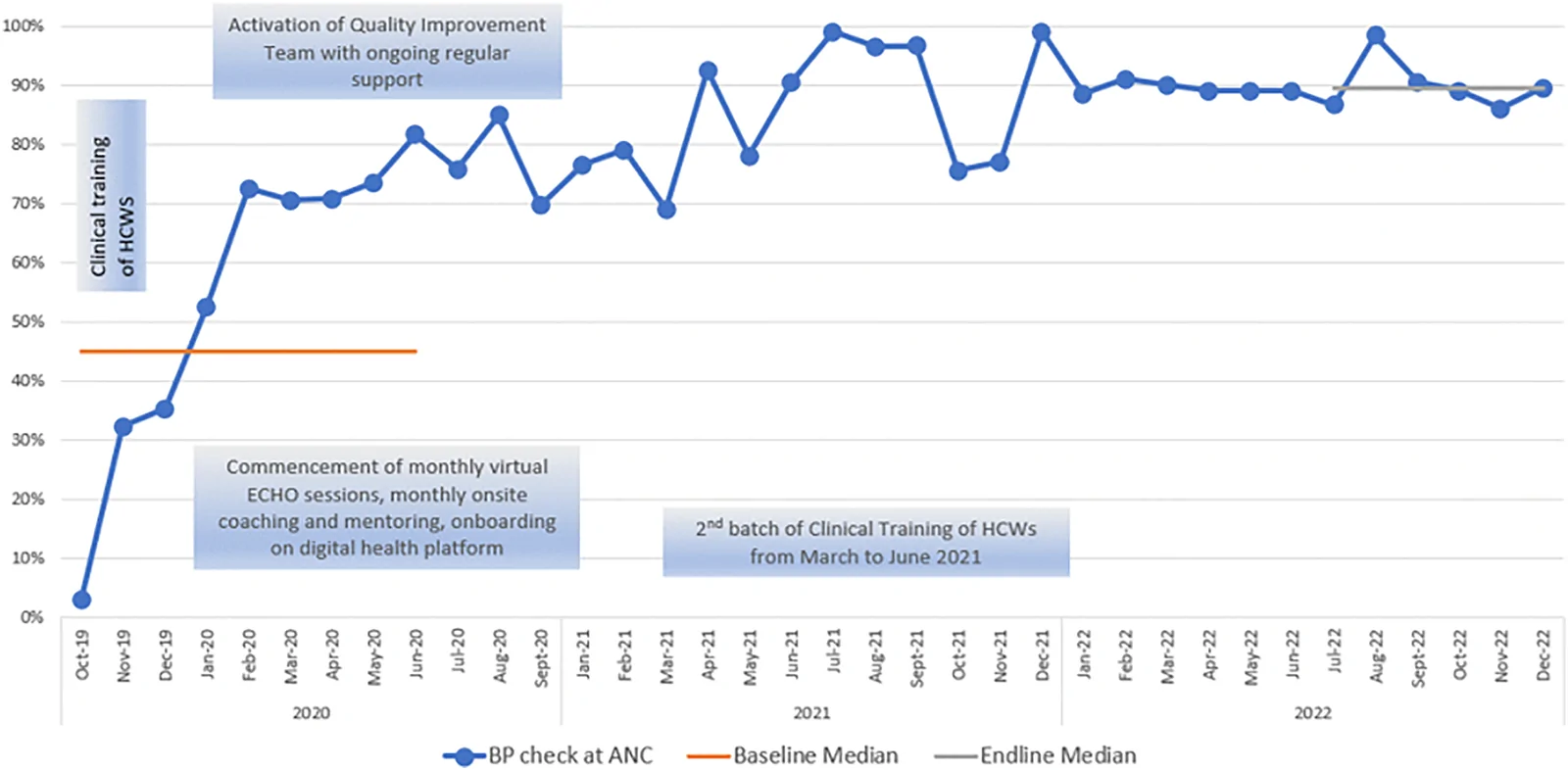
Percentage of ANC visits with blood pressure documented to assess for HDP in Lagos and FCT (n = 93,384 ANC visits in 20 facilities 2019-2020 and in 40 health facilities 2021-2022).

Among 93,384 ANC visits across the 40 program facilities from October 2019-December 2022, the median proportion of women with a documented blood pressure check increased from 44% at baseline in 20 facilities (October 2019-March 2020) to 89% in the final program 6 months in 40 facilities (July – December 2022). The median proportion of women checked for anemia and diabetes during ANC visits in the 40 facilities increased from 48% to 91% and from 32% to 87%, respectively during the same baseline and endline periods. Analysis of run chart results for each of the 3 ANC indicators (Figs 6–8) demonstrates a shift of at least 6 consecutive data points above the baseline median value (initial 6 data points for each ANC indicator) suggesting that the measured improvements were due to the program interventions and improvements being implemented by the QI teams in each facility.

#### Documentation of blood pressure check during Postnatal Care and Family Planning visits among women of reproductive age in 40 program facilities (2021–2022).

**Fig 9:**
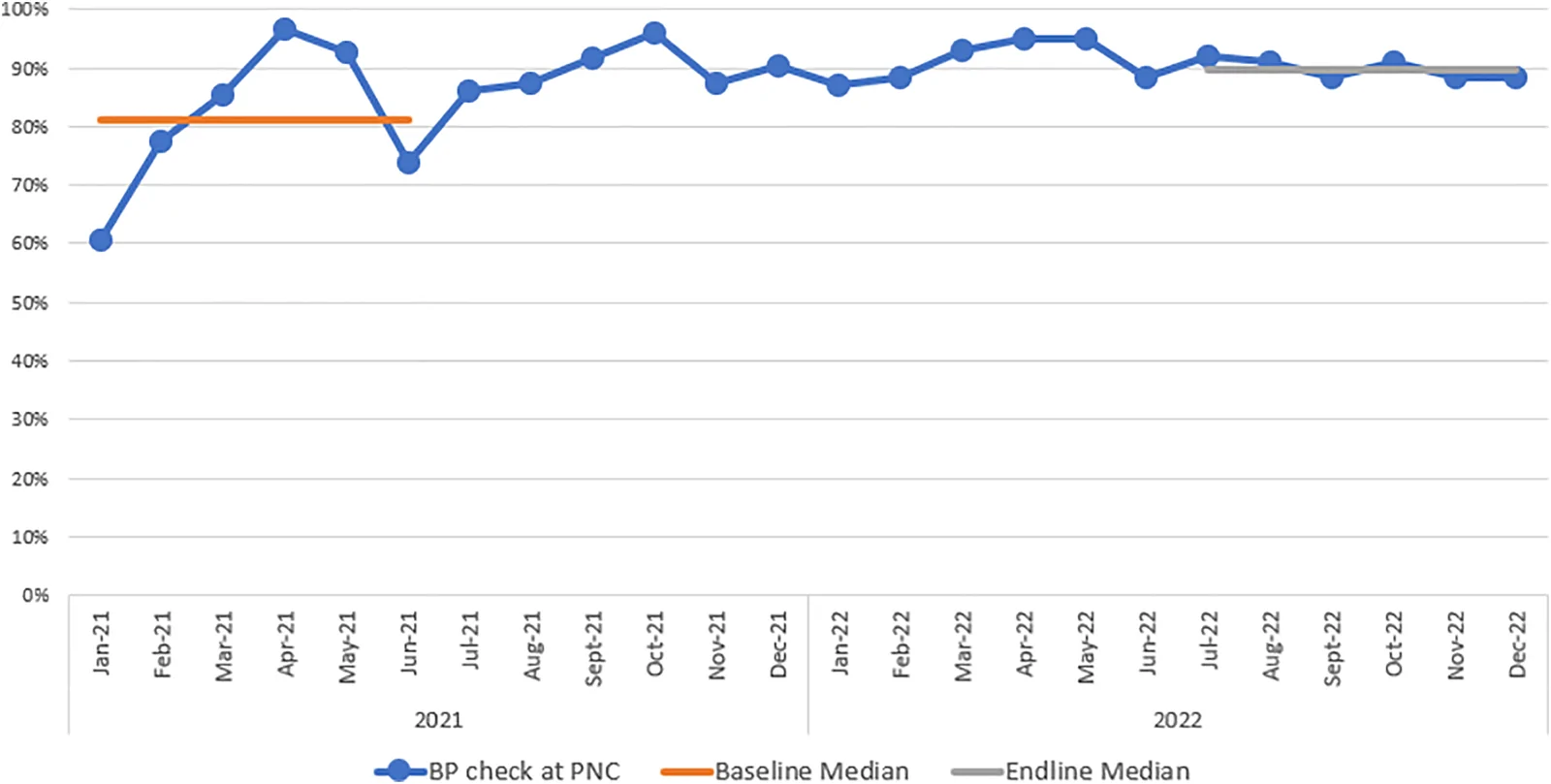
Proportion of PNC visits with a documented blood pressure check (n = 57,658 PNC visits in 40 health facilities in Lagos & FCT, 2021 to 2022).

**Fig 10:**
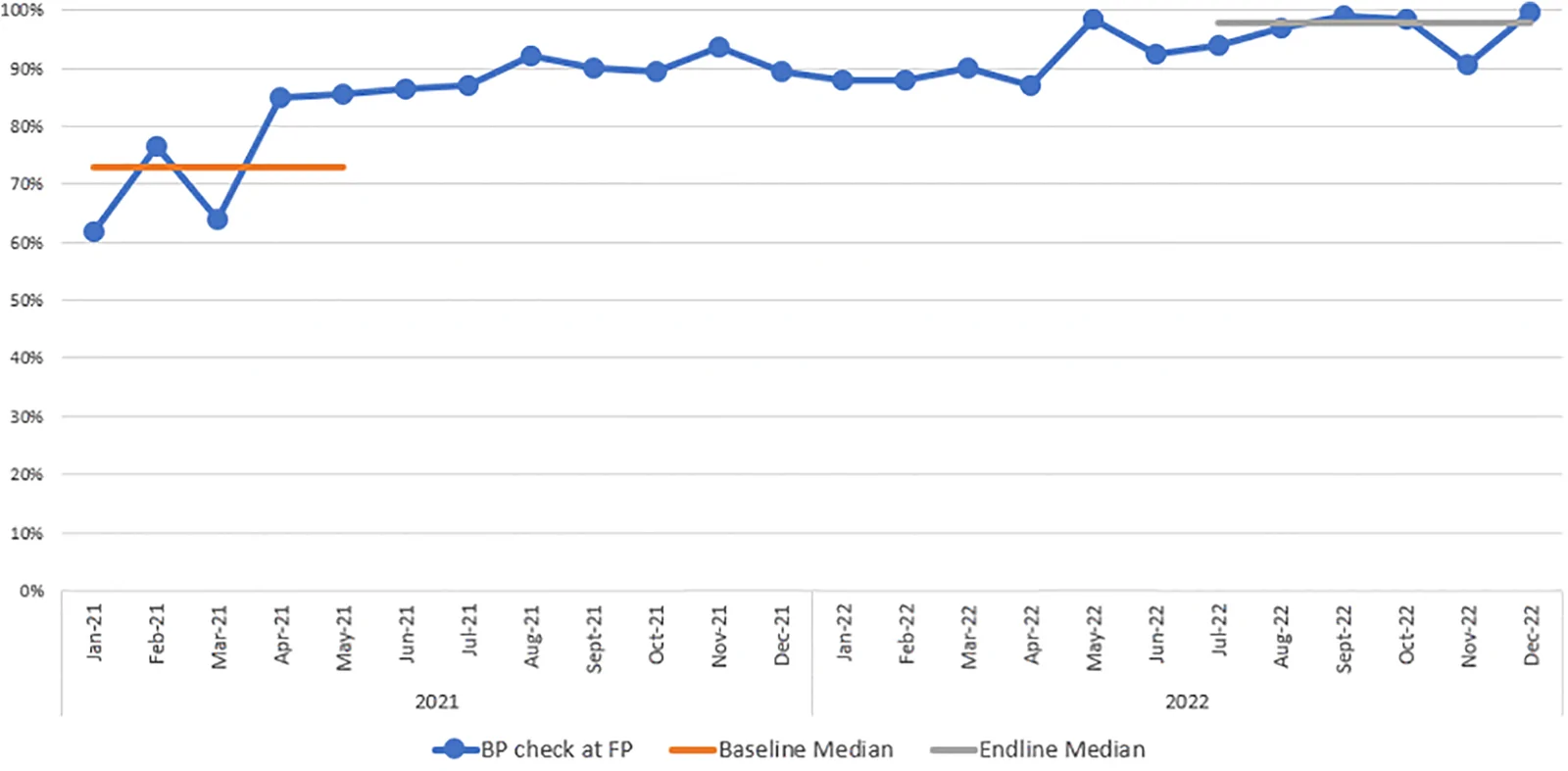
Proportion of FP visits with a documented blood pressure check (n = 105,237 FP visits in 40 health facilities in Lagos & FCT – 2021 to 2022).

Among 57,658 Postnatal Care (PNC) visits in the 40 program facilities, the median proportion of visits with a documented blood pressure check increased from 81% at baseline (January-June 2021) to 91% during the final program 6 months (July-December 2022). Among 105,237 Family Planning (FP) visits, the median proportion of visits with a documented blood pressure increased from 73% to 98% in the 40 program facilities during the same baseline and endline periods (Figs 9 and 10).

## Discussion

This case study of a multi-component program to improve early detection of HDP and risk factors among women attending maternal and reproductive health services in a setting with known high prevalence of HDP and associated mortality demonstrated sustained improvements in assessment for HDP and risk factors (anemia, diabetes) during routine ANC, PNC and FP visits in 40 health care facilities in Lagos State and the Federal Capital Territory in Nigeria.

Results of the program’s facility ANC assessment demonstrate important gaps in providers’ knowledge and self-reported confidence and in observed provider adherence with best practices for identification of HDP and risk factors during ANC visits. These findings are consistent with prior studies from Nigeria and other sub-Saharan African settings documenting low provider knowledge for HDP management in maternal health services, including among midwives [24–28] A cross-sectional analysis of the quality of facility ANC services for detecting and managing HDP in seven states in Nigeria demonstrated significant gaps in provider knowledge comparable to our findings, including low knowledge of HDP symptoms, HDP diagnosis, management of PE/E and correct use of antihypertensive drugs [29]. Comparable to our program’s ANC assessment findings, in which only 38% of ANC providers asked about a personal history of PE/E, only 50% of observed ANC visits in 95 facilities in this analysis asked about a pregnant woman’s past medical history. The failure to ask about a pregnant woman’s medical history, including a history of PE, during ANC visits represents a critical missed opportunity to identify and initiate appropriate management in pregnant women with elevated HDP risk (such as initiating aspirin).

The program met its objective of strengthening HCW knowledge of best practices for identification and management of HDP and risk factors. Baseline program ANC assessment findings measured low levels of provider knowledge and self-reported confidence related to safe antihypertensive use in pregnancy, diabetes management, and interpretation of body mass index—gaps that have been previously documented in similar settings [20,26,27,30,31]. Post-training improvements in knowledge, with most HCWs achieving knowledge scores greater than 80%, suggest that the program training intervention was effective in improving provider knowledge. The Federal Ministry of Health Task-shifting and Task-sharing Policy for Essential Health Care Services in Nigeria and a recent Hypertension Academy training program in Nigeria offer important policy and training platforms to strengthen knowledge and skills of midwives, Community Health Extension Workers (CHEWS) and other providers in the Nigeria setting to improve assessment, identification and management of HDP and risk factors during maternal and reproductive health services and as part of ongoing primary care for women at risk for premature cardiovascular disease due to a history of HDP [32].

The translation of knowledge into sustained practice changes was likely facilitated by ongoing program interventions including clinical mentoring of providers and coaching of facility QI teams which helped address provider uncertainty, reinforce correct practices, and address underlying constraints that impede integration of HDP best practices into routine ANC, PNC and FP visit workflows [30,31,33]. Beyond confirming existing evidence for weak adherence with HDP best practices, the ANC assessment and the ongoing work of the QI teams in program facilities helped explain why gaps persist—namely weak integration of NCD screening into maternal health workflows, inadequate clinical decision support, inconsistent documentation practices, and limited use of routine data for quality improvement. These system-level constraints were an important focus of the program QI interventions. Facility QI teams were supported to analyse and address root causes for poor adherence with HDP best practices and to use routine health information data to monitor and help guide changes to improve HDP and risk factor best practices as part of routine ANC, PNC and FP visit workflows.

Key program interventions included initial onsite competency-based clinical and QI capacity strengthening complemented by monthly virtual training sessions to reinforce health care workers (HCWs) knowledge, and the activation and ongoing support of health facility quality improvement teams (QIT) complemented by digital self-care support of women with HDP risk factors via enrollment onto a digital health platform. The sustained upward shifts observed in the ANC, PNC and FP indicator run charts suggest that improvements were not driven by clinical capacity building alone but reinforced through continuous QI processes, including routine data review, and regular peer learning. Evidence from systematic reviews indicates that QI approaches that combine team-based problem solving with data use are more effective in improving provider practices in low- and middle-income countries than training or supervision alone [34,35]. The findings from this case study are consistent with this evidence and highlight the added value of embedding QI structures within routine maternal and reproductive health services.

Notably, the program demonstrated improvements in screening for HDP and risk factors not only during ANC but also during PNC and FP visits—platforms that are often underutilized for NCD and HDP risk factor identification despite their importance for reaching women beyond pregnancy [13,25]. In Nigeria, where ANC attendance is limited and many women have limited contact with the health system outside pregnancy, leveraging FP and PNC services expands opportunities for early identification of HDP and risk factors. Our program demonstrates the feasibility of integrating HDP and risk factor screening beyond pregnancy into routine reproductive health services to reach more women and reduce missed opportunities to identify women with HTN, HDP and risk factors at critical health care touch points. The higher proportion of PNC and FP visits incorporating assessment of BP at baseline (in 2021) is likely due to the earlier program interventions from October 2019–2020 to improve BP assessment during ANC visits in these same facilities.

Improving routine screening for HDP and risk factors at key service delivery maternal, reproductive and primary health care touch points is an important strategy to reach more women of reproductive age with timely diagnosis and prevention and management of HDP and risk factors to reduce maternal and perinatal mortality and reduce premature cardiovascular disease. Although our program did not assess downstream clinical outcomes such as blood pressure control, treatment adherence, or maternal and perinatal complications, the measured improvements in assessment for HDP and risk factors as part of routine ANC, PNC and FP visits facilitated more timely identification and linkages to care for women with previously undetected HDP and risk factors (program training and mentoring interventions included appropriate management of HDP and risk factors in women of reproductive age). Early identification is particularly important given the established association between HDP, anemia, diabetes, and the increased risk for maternal mortality, adverse perinatal outcomes, and long-term cardiovascular disease in affected women [13,14,17,22].

The program findings also contribute to the theoretical discourse on integrating NCD and maternal health services. This case study supports the optimization of maternal and reproductive health platforms as important entry points for addressing NCD risk and diagnosis among WRA, rather than as isolated services focused exclusively on pregnancy outcomes [36]. By embedding HDP, diabetes and anemia screening within existing maternal and reproductive health services, our program demonstrates a pragmatic approach for addressing the epidemiological transition influencing direct and indirect maternal deaths in Nigeria without creating parallel systems of care.

More work is needed to improve clinical management and self-care support for WRA with HDPs and NCDs, including continuity and coordination of care across time, service type (ANC, birth, PNC, FP and PHC services) and system levels (primary and secondary). At the policy and program level it is important to strengthen linkages and coordination across maternal health (MH), FP, PHC and NCD programs and to include WRA and pregnant women in existing NCD and PHC programs.

Limitations of our program include the absence of routine monitoring of clinical outcomes and adherence to HDP and risk factor management protocols, which weakens conclusions regarding the potential impact of improved screening on health outcomes. Implementation challenges—including staff turnover, equipment shortages, gaps in routine health information systems, and service disruptions during the COVID-19 pandemic—may also have affected program fidelity and limit generalizability. Nonetheless, the consistency of improvements across facilities and service delivery platforms suggests that the program was feasible and resilient within real-world health system constraints. Overall, our program case study provides practical evidence on feasible strategies to strengthen efforts at prevention and early detection of HDP and risk factors as part of routine maternal and reproductive health services in a high-burden setting.

### Implications for future research and program

Further research and program learning is needed to determine whether similar program interventions lead to improvements in identification and management of HDP and risk factors and maternal and perinatal outcomes. Future studies incorporating monitoring of health care processes and outcomes and comparative designs would strengthen evidence of effectiveness. Program interventions and findings suggest that sustainability and scale are more likely when QI team structures, indicator tracking, and mentoring mechanisms are embedded within routine facility and government quality systems rather than implemented as project-specific activities. Institutionalizing these elements within existing maternal and reproductive health programs will support continuity and broader adoption of the program interventions.

### Conclusion

This case study demonstrates that a multi-component program intervention implemented through routine ANC, PNC, and FP services can significantly improve screening for hypertension, anemia, and diabetes among women of reproductive age in two Nigerian states. Program results demonstrate that combining competency-based training with facility-level quality improvement processes and supportive mentoring is associated with sustained improvements in provider practices. Embedding screening activities across multiple service touchpoints expanded opportunities for identifying women at risk of HDP and related non-communicable diseases, including beyond pregnancy alone.

Although clinical outcomes were not assessed, the demonstrated gains in screening coverage represent an important foundation for improving identification and management of HDP and risk factors. Program findings provide contextually relevant learning for integration of HDP and NCD prevention and early detection into maternal and reproductive health services in similar resource-constrained settings.

## Recommendations

1 Adopt multi-component quality improvement approaches rather than training-only models.
2 Design an experimental study to build evidence of effectiveness and feasibility of the program interventions for improving early detection and management of HDP and risk factors at key service delivery touchpoints for women of reproductive age
3 Advocate for inclusion and monitoring of service delivery HDP and risk factor data elements in routine health information systems to optimize detection and control of HDP and risk factors in maternal health and primary health care services
4 Strengthen facility-based quality improvement teams and data-use practices to improve early detection and management of HDP and risk factors among WRA to reduce maternal mortality and premature cardiovascular disease

## Supporting information

S1 Data Dataset. (ZIP)
