# Correction: Addressing risk factors for hypertensive disorders in pregnancy: A case study of a quality improvement program in two states in Nigeria

**DOI:** 10.1371/journal.pone.0362798

**Published:** 2026-11-10

**Authors:** Ugo Okoli, Sylverius Obafemi, Comfort Okpe, Kendra Njoku, Nneka Mobisson, Chinyere Uzoama, Victoria Omoera, Emmanuel Ugwa, Kathleen Hill

The Funding statement for this article is incorrect. The correct Funding statement is as follows: This study was financially supported by MSD for Mothers in the form of a grant awarded to and administered to the authors by Jhpiego Corporation. No additional external funding was received for this study. The funders had no role in study design, data collection and analysis, decision to publish, or preparation of the manuscript.

The Competing Interests statement for this article is incorrect. The correct Competing Interests statement is as follows: The authors have read the journal's policy and have the following competing interests: SO, UO, CO, and KH are employees of Jhpiego Corporation. The funder, MSD for Mothers, is an initiative of Merck & Co Inc. of Kenilworth, NJ. This does not alter our adherence to PLOS ONE policies on sharing data and materials. There are no patents, products in development or marketed products associated with this research to declare.
