# Supplementary material for: Addressing risk factors for hypertensive disorders in pregnancy: A case study of a quality improvement program in two states in Nigeria
Source: PLoS One. 2026 Sep 3;21(9):e0356879. doi: 10.1371/journal.pone.0356879 (PMC13541259; doi:10.1371/journal.pone.0356879)
Supplement: Supplementary file 1 [file pone.0356879.s001.zip › Dataset/Healthcare worker Survey Questionnaire _ REDCap.pdf]

Data Dictionary Codebook

05/01/2025 4:03am

#	Variable / Field Name	Field Label<br>*Field Note*	Field Attributes (Field Type, Validation, Choices, Calculations, etc.)
**Instrument: Health Worker Survey (health_worker_survey)**			
1	[record_id]	Record ID	text
2	[study_title]	Study Title: Assessment of the prevalence of indirect causes (Hypertension, Diabetes, Anemia, Obesity) of maternal mortality associated with pre-eclampsia/eclampsia (PE/E) and quality of care for prevention and management of these PE/E risk factors across the maternal and reproductive health care continuum in Nigeria.<br>Principal Investigator: Dr Chibugo (Ugo) Okoli<br>IRB No.: 00009452<br>PI Version 2, Date: 04/11/19	descriptive
3	[latitude]	GPS Coordinates: Latitude	text, Required<br>Custom alignment: RH<br>Field Annotation: @LATITUDE
4	[longitude]	GPS Coordinates: LONGITUDE	text, Required<br>Custom alignment: RH<br>Field Annotation: @LONGITUDE
5	[instruction1]	BEFORE THE INTERVIEW/SURVEY, OBTAIN PERMISSION FROM THE PARTICIPANT. MAKE SURE THAT THE PARTICIPANT KNOWS TH	descriptive
6	[liketojoin]	Section Header: *Hello, I am ________________. I am representing Jhpiego, Johns Hopkins University affiliate and would like to talk to you about a study we are conducting to assess the prevalence of specified indirect causes (hypertension, diabetes mellitus, anemia and obesity) of maternal morbidity and mortality linked to pre-eclampsia/eclampsia (PE/E) and to assess the quality of care for prevention, diagnosis and management of these non-communicable diseases (NCDs) in four LGAs in Lagos State and the Federal Capital Territory (FCT). This study is part of the RH/MH-NCD Quality of Care Project supported by MSD for Mothers. • We are interested in learning about your knowledge and practice of these NCD services in the care of women within the MH/RH continuum in this facility. • We ask you to participate in this study because you provide MH/RH healthcare services in this facility. • If you say yes, we will ask you to we will ask you to participate in a survey. The questionnaire for the survey will be self-administered and it may take up to one hour. • You may feel uncomfortable or embarrassed answering some of the questions. You are free not to answer any of the questions in the questionnaire, and you can stop your participation in the study at any time. Although we would appreciate your participation, you are free to decide not to participate. • You do not have to answer all the questions and you may stop at any time. The information acquired from this survey may be used by the Ministry of Health or other organizations to improve services, or for research on health services. You do not have to agree to be part of this study, and you may change your mind at any time. • There is a risk that someone outside the study will see your information. We will do our best to keep your information safe by de-identifying any personal identifiers using a special code and locking up in a safe and secure cabinet. When we share your information with other researchers, we will ask them to use the same protections. • There may be no direct benefit to you from being in this study; you may get satisfaction from knowing that in the future, the study findings may contribute to efforts to improve maternal health services. • We will let the community/state know about the results of the study. • Do you have any questions? [Probe to assess the participant's understanding.]*<br>II. Would you like to join the study?	radio, Required<br>1 Yes<br>0 No
7	[consent]<br>Show the field ONLY if:<br>[liketojoin] = '1'	Section Header: *You may call the lead local investigator, Dr. Chibugo Okoli (08031973001) or Dr. Oluwatobi Adebayo (08135405023), if you have questions or complaints about being in this study. If you have any questions about your rights as a research participant, you may also contact the local Ethics Committee which approved this study about any problems or concerns at: Chairman@nhrec.net. Telephone: 08035868293 OR Secretary@nhrec.net. Telephone: 08036785071.*<br>Do I have your permission to continue?	radio, Required<br>1 Yes, consent is given<br>0 No, consent is not given
8	[interviewer_sign]<br>Show the field ONLY if:<br>[consent] = '1'	Interviewer's Signature and date: (indicates respondent's willingness to participate):	file (signature)
9	[instruction_continue]<br>Show the field ONLY if:<br>[consent] = '1'	Your responses are completely anonymous and confidential and will never be linked to you individually. It should take about 30-40 minutes to complete the questionnaire. We greatly appreciate your time.	descriptive

10	[name_state]<br>Show the field ONLY if:<br>[consent] = '1'	Name of State where Health Facility is Located	radio<br>1 Abuja<br>2 Lagos
11	[abuja_lga_name]<br>Show the field ONLY if:<br>[name_state] = '1'	Name of LGA where Health Facility is Located in ABUJA	radio<br>1 Abuja Municipal Area Council<br>2 BWARI
12	[lagos_lga_name]<br>Show the field ONLY if:<br>[name_state] = '2'	Name of LGA where Health Facility is Located in LAGOS	radio<br>3 Alimosho<br>4 Ikorodu
13	[hfname_abuja]<br>Show the field ONLY if:<br>[abuja_lga_name] = '2' or [abuja_lga_name] = '1'	Facility Name_ABUJA	dropdown<br>1 Wuse GH<br>2 Nyanya GH<br>3 PHC Gidan Mangoro<br>4 PHC Lugbe<br>5 Salvon Hospital<br>6 Bwari GH<br>7 Kubwa GH<br>8 Dutse Alhaji PHC<br>9 Mpape PHC<br>10 Daughters of Charity Catholic Mission
14	[hfname_lagos]<br>Show the field ONLY if:<br>[lagos_lga_name] = '3' or [lagos_lga_name] = '4'	Facility Name _ LAGOS	dropdown<br>11 Alimosho GH<br>12 Ipaja PHC<br>13 Akowonjo PHC<br>14 Rauf Aregbesola<br>15 Mobonike Hospital<br>16 Ikorodu GH<br>17 Ita Elewa phc<br>18 Odonla PHC<br>19 Oke Eletu PHC<br>20 Oak Hospital
15	[facility_type]<br>Show the field ONLY if:<br>[consent] = '1'	Facility Type	radio<br>1 District/General hospital<br>2 Mission Hospital<br>3 Private Hospital<br>4 Primary health center
16	[hf_carelevel]<br>Show the field ONLY if:<br>[consent] = '1'	Health Facility Level of Care	radio<br>1 Tertiary Level of Care<br>2 Secondary Level of Care<br>3 Primary Level of Care
17	[q1]<br>Show the field ONLY if:<br>[consent] = '1'	Section Header: *Section A: Background Information*<br>Q1. Please indicate the type of health care provider you are:<br>*If Specialist Physician, please specify*	radio<br>1 Generalist (non-specialist) medical doctors<br>2 Specialist medical doctors<br>3 Pharmacists<br>4 Laboratory technicians (medical and pathology)<br>5 Community health officer<br>6 Community Health Extension Workers<br>7 Midwife<br>8 Nurse<br>9 Nurse/Midwife<br>10 Medical Officer/Generalist physician<br>11 Specialist Physician

18	[q1a]<br><br>Show the field ONLY if:<br>[q1] = '11'	Q1a. If Specialist Physician, please specify	radio<br>1 Obstetrician-Gynecologist<br>2 Endocrinologist<br>3 Cardiologist<br>95 Other (please specify)
19	[q1ai]<br><br>Show the field ONLY if:<br>[q1a] = '95'	Q1aI. Other, specify	notes
20	[q2]<br><br>Show the field ONLY if:<br>[consent] = '1'	Q2. Please indicate your gender<br>*Request the respondent to indicate his/her gender*	radio<br>1 Female<br>2 Male
21	[q3]<br><br>Show the field ONLY if:<br>[consent] = '1'	Q3. How many years has it been since you completed your professional training?	radio<br>1 < 5 years<br>2 5-10 years<br>3 10-20 years<br>4 > 20 years
22	[q4]<br><br>Show the field ONLY if:<br>[consent] = '1'	Q4. Do you provide care for pregnant women?	radio<br>1 Yes<br>0 No
23	[q5]<br><br>Show the field ONLY if:<br>[q4] = '1'	Q5. If yes, please check which apply; otherwise skip to next question<br>*If yes to the previous question*	checkbox<br>1 q5___1 I provide routine ANC<br>2 q5___2 I see pregnant women who develop a complication and need more specialized care (e.g. high blood pressure, diabetes, severe anemia)<br>95 q5___95 Other (please specify)
24	[q5a]<br><br>Show the field ONLY if:<br>[q4] = '1'	Q5a. How long have been working in this unit?	radio<br>1 Less than 6 months<br>2 6 months<br>3 Greater than 6 months
25	[q6]<br><br>Show the field ONLY if:<br>[consent] = '1'	Section Header: *Check List for Post Pregnancy and Maternal Activities*<br>Q6. Do you provide childbirth services?	radio (Matrix)<br>1 Yes<br>0 No<br><br>Field Annotation: Select single option
26	[q7]<br><br>Show the field ONLY if:<br>[consent] = '1'	Q7. Do you provide postnatal care for women during the first two months after birth?	radio (Matrix)<br>1 Yes<br>0 No<br><br>Field Annotation: Select single option
27	[q8]<br><br>Show the field ONLY if:<br>[consent] = '1'	Q8. Do you provide family planning services for women?	radio (Matrix)<br>1 Yes<br>0 No<br><br>Field Annotation: Select single option
28	[q9]<br><br>Show the field ONLY if:<br>[consent] = '1'	Q9. Do you provide care for women with acute illnesses (e.g. cough, fever)?	radio (Matrix)<br>1 Yes<br>0 No<br><br>Field Annotation: Select single option
29	[q10]<br><br>Show the field ONLY if:<br>[consent] = '1'	Q10. Do you provide ongoing care for women with chronic conditions e.g. Hypertension, Diabetes?	radio (Matrix)<br>1 Yes<br>0 No<br><br>Field Annotation: Select single option

30	[q11]<br>Show the field ONLY if:<br>[consent] = '1'	Section Header: *Section B: Hypertension Please answer the questions below as best as you can. If you do not know the correct response, there is no problem. Section B: In this section I will ask you a few questions on your knowlegde of hypertension(Introduce all sections and subsections, highlight 'bold')*<br>Q11. Is there a blood pressure apparatus (may be digital or manual sphygmomanometer) in this facility/unit today?	radio<br>1 Yes<br>0 No
31	[q12]<br>Show the field ONLY if:<br>[consent] = '1'	Q12. Is the blood pressure currently functional?	radio<br>1 Yes<br>0 No
32	[q13]<br>Show the field ONLY if:<br>[consent] = '1'	Q13. Is there a Stethoscope) in this facility/unit today?	radio<br>1 Yes<br>0 No
33	[q14]<br>Show the field ONLY if:<br>[consent] = '1'	Q14. Is it currently functional?	radio<br>1 Yes<br>0 No
34	[q15]<br>Show the field ONLY if:<br>[consent] = '1'	Q15. How long have you been measuring blood pressure?	radio<br>1 Less than 3 years<br>2 3 - 6 years<br>3 7 - 10 years<br>4 11 - 14 years<br>5 More than 15 years
35	[q16]<br>Show the field ONLY if:<br>[consent] = '1'	Q16. Do you have any hearing problems?	radio<br>1 Yes<br>0 No
36	[q17]<br>Show the field ONLY if:<br>[consent] = '1'	Q17. Have you had your hearing checked in the last 3 years?	radio<br>1 Yes<br>0 No
37	[q18]<br>Show the field ONLY if:<br>[consent] = '1'	Q18. Do you have any visual problems?	radio<br>1 Yes<br>0 No
38	[q19]<br>Show the field ONLY if:<br>[consent] = '1'	Q19. Have you had your eyes checked in the last 3 years?	radio<br>1 Yes<br>0 No
39	[q20]<br>Show the field ONLY if:<br>[consent] = '1'	Q20. Do you have the guidelines for blood pressure measurement?	radio<br>1 Yes<br>0 No
40	[q21]<br>Show the field ONLY if:<br>[consent] = '1'	Q21. Have you read the guidelines for blood pressure measurement recently?	radio<br>1 Yes<br>0 No
41	[q22]<br>Show the field ONLY if:<br>[consent] = '1'	Q22. When did you last receive training on blood pressure measurement technique?	radio<br>1 Less than a year<br>2 1 year<br>3 2 years<br>4 3 years<br>5 4 years<br>6 5 years<br>7 6 - 10 years<br>8 More than 10 years<br>0 Never
42	[q23]<br>Show the field ONLY if:<br>[consent] = '1'	Q23. Which of the following factors will have an influence on blood pressure?	checkbox<br>1 q23___1 Full urinary bladder<br>2 q23___2 Coffee 30 minutes prior to BP reading<br>3 q23___3 Small cuff on a large arm\<br>4 q23___4 All of the above

43	[q24]<br>Show the field ONLY if:<br>[consent] = '1'	Q24. Which arm should be used to measure BP?	radio<br>1 Left arm<br>2 Right Arm<br>3 Bilateral arms
44	[q25]<br>Show the field ONLY if:<br>[consent] = '1'	Q25. Do you record the specific arm when BP is charted?	radio<br>1 Always<br>2 Sometimes<br>0 Never
45	[q26]<br>Show the field ONLY if:<br>[consent] = '1'	Q26. Are there any circumstances when it might be appropriate to measure the BP in both arms?	radio<br>1 Yes<br>0 No
46	[q27]<br>Show the field ONLY if:<br>[consent] = '1'	Q27. Do you expect the BP to be the same in both arms?	radio<br>1 Yes<br>0 No<br>97 Don't Know
47	[q28]<br>Show the field ONLY if:<br>[consent] = '1'	Q28. For how long should a patient rest before BP measurement?	radio<br>1 5 minutes<br>2 Less than 5 Minutes<br>3 Greater than 5 minutes<br>4 Unnecessary to rest
48	[q29]<br>Show the field ONLY if:<br>[consent] = '1'	Q29. How long should you wait in between two consecutive BP readings on the same individual?	radio<br>1 2 minutes<br>2 5 minutes<br>3 10 minutes<br>4 More than 10 minutes<br>5 No specific time between measurements required
49	[q30]<br>Show the field ONLY if:<br>[consent] = '1'	Q30. What should the position of the subject's arm be during BP measurement?	radio<br>1 Under heart level<br>2 Above heart level<br>3 At heart level
50	[q31]<br>Show the field ONLY if:<br>[consent] = '1'	Q31. Should the arm be supported during measurement?	radio<br>1 Yes, by the nurse<br>2 Yes, by a surface<br>0 Unsupported
51	[q32]<br>Show the field ONLY if:<br>[consent] = '1'	Q32. Should the elbow be slightly flexed or straight?	radio<br>1 Slightly flexed<br>2 Straight<br>97 Don't know
52	[q33]<br>Show the field ONLY if:<br>[consent] = '1'	Q33. Do you access the subject's arm for cuff size before applying a cuff?	radio<br>1 Always<br>2 Sometimes<br>0 Never
53	[q34]<br>Show the field ONLY if:<br>[consent] = '1'	Q34. What cuff size would cause the greatest error?	radio<br>1 Narrow cuff<br>2 Wide cuff<br>97 Don't know
54	[q35]<br>Show the field ONLY if:<br>[consent] = '1'	Q35. Do you palpate to identify the systolic pressure before using the stethoscope?	radio<br>0 Never palpated<br>1 Sometimes<br>2 Always

55	[q36]<br>Show the field ONLY if:<br>[consent] = '1'	Q36. What is the reason for identifying systolic pressure by palpation?	radio<br>1 To get a more accurate reading<br>2 To avoid over inflation<br>3 To identify where to place the stethoscope<br>97 Don't know
56	[q37]<br>Show the field ONLY if:<br>[consent] = '1'	Q37. Which of the following statements is/are correct about the definition of high blood pressure in a pregnant woman:	radio<br>1 Systolic BP > 150; diastolic BP > 85<br>2 Systolic BP > 135; diastolic BP > 90<br>3 Systolic BP > 140; diastolic BP> 90<br>4 Systolic BP > 145; diastolic BP > 95<br>97 Do not know
57	[q38]<br>Show the field ONLY if:<br>[consent] = '1'	Q38. A woman who has a a family history of hypertension is at higher risk of hypertension in pregnancy/preeclampsia?	radio<br>1 True<br>0 False
58	[q39]<br>Show the field ONLY if:<br>[consent] = '1'	Q39. High blood pressure increases risk for pre-eclampsia during pregnancy or in future pregnancies	radio<br>1 True<br>2 False
59	[q40]<br>Show the field ONLY if:<br>[consent] = '1'	Q40. A woman who has a history of pre-eclampsia is at higher risk for pre-eclampsia during a future pregnancy	radio<br>1 Yes<br>0 No
60	[q41]<br>Show the field ONLY if:<br>[consent] = '1'	Q41. A woman with a history of pre-eclampsia is at higher risk for developing future cardiovascular disease (high blood pressure, heart disease, stroke):	radio<br>1 True<br>0 False
61	[q42]<br>Show the field ONLY if:<br>[consent] = '1'	Q42. Preelampsia can affect the health of both mother and fetus?	radio<br>1 True<br>0 False
62	[q43]<br>Show the field ONLY if:<br>[consent] = '1'	Q43. Hypertensive disorders of pregnancy include which of the following:	radio<br>1 Pregnancy-induced HTN(gestational hypertension);<br>2 White-coat hypertension;<br>3 Chronic HTN;<br>4 Intermittent high blood pressure<br>5 Pre-eclampsia/; eclampsia
63	[q44]<br>Show the field ONLY if:<br>[consent] = '1'	Q44. A pregnant woman with high blood pressure should be treated with an antihypertensive if her blood pressure is not controlled with diet and exercise:	radio<br>1 True<br>0 False
64	[q45]<br>Show the field ONLY if:<br>[consent] = '1'	Q45. A beta blocker antihypertensive medication (e.g. propanolol) is safe to use in pregnancy:	radio<br>1 True<br>0 False
65	[q46]<br>Show the field ONLY if:<br>[consent] = '1'	Q46. A calcium channel blocker antihypertensive medication (e.g. Amlodipine) is safe to use in pregnancy:	radio<br>1 True<br>0 False
66	[q47]<br>Show the field ONLY if:<br>[consent] = '1'	Q47. An angiotensin-converting enzyme inhibitor (ACEI) e.g. lisinopril is safe to use in pregnancy	radio<br>1 True<br>0 False
67	[q48]<br>Show the field ONLY if:<br>[consent] = '1'	Q48. Which statement is correct regarding common symptoms of pre-eclampsia?	radio<br>1 Abdominal pain, palpitations, headache<br>2 Headache, abdominal pain, blurred vision<br>3 Urinating more frequently, headache, blurred vision<br>4 Headache, palpitations, chest pain

68	[q49]<br>Show the field ONLY if:<br>[consent] = '1'	Q49. Pre-eclampsia may commonly present (for the first time) after a woman gives birth during the early postnatal period:	radio<br>1 True<br>0 False
69	[q50]<br>Show the field ONLY if:<br>[consent] = '1'	Q50. A woman diagnosed with high blood pressure or pre-eclampsia during pregnancy, childbirth or the postnatal period should be seen for follow-up on a regular basis to monitor her blood pressure:	radio<br>1 True<br>0 False
70	[section_c]<br>Show the field ONLY if:<br>[consent] = '1'	Section C: In this section I will ask you a few questions on your knowlegde of diabetes mellitus	descriptive
71	[q51]<br>Show the field ONLY if:<br>[consent] = '1'	Q51. Which of the following is correct about the definition of Diabestes Mellitus in a pregnant woman:	checkbox<br>1 q51___1 Oral Glucose Tolerance Test (OGTT) ≥ 140 mg/dL<br>2 q51___2 OGTT ≥ 7.8 mmol/L<br>3 q51___3 OGTT= 140mg/dL<br>0 q51___0 None of the above<br>97 q51___97 Don't know
72	[q52]<br>Show the field ONLY if:<br>[consent] = '1'	Q52. A woman who has a a family history of Diabestes Mellitus is at higher risk of gestational Diabestes Mellitus?	radio<br>1 True<br>0 False
73	[q53]<br>Show the field ONLY if:<br>[consent] = '1'	Q53. Diabetes increases risk for pre-eclampsia during pregnancy or in future pregnancies	radio<br>1 True<br>0 False
74	[q54]<br>Show the field ONLY if:<br>[consent] = '1'	Q54. Diabetes can affect the health of both mother and fetus?	radio<br>1 True<br>0 False
75	[q55]<br>Show the field ONLY if:<br>[consent] = '1'	Q55. Which option is correct regarding common symptoms of diabetes:	radio<br>1 Blurred vision; ankle swelling; palpitations; abdominal pain<br>2 Thirsty all the time (drinks a lot); increased urination; weight loss; blurred vision<br>3 Headache; abdominal pain; thirsty all the time; increased urination<br>4 Weight loss; palpitations; headache; ankle swelling
76	[q56]<br>Show the field ONLY if:<br>[consent] = '1'	Section Header: *Section E: In this section I will ask you a few questions on your knowlegde of anemia*<br>Q56. Is there a functional equipment or means of assessing client's b;ood level (Hb or PCV) today?<br>*If Yes please specify*	radio<br>1 Yes<br>0 No
77	[q56a]<br>Show the field ONLY if:<br>[consent] = '1'	Q56a. If yes to the previous question please specify.	notes
78	[q57]<br>Show the field ONLY if:<br>[consent] = '1'	Q57. How often do you estimate a client's blood level?<br>*Please specify.. Write 97 for do no know*	notes
79	[q58]<br>Show the field ONLY if:<br>[consent] = '1'	Q58. What are the criteria for diagnosis of anemia in pregnancy as per national guidelines?<br>*Please specify.. Write 97 for do no know*	notes
80	[q59]<br>Show the field ONLY if:<br>[consent] = '1'	Q59. Which are the criteria for diagnosis of anemia in non-pregnant women as per national guidelines:<br>*Please specify.. Write 97 for do no know*	notes
81	[q60]<br>Show the field ONLY if:<br>[consent] = '1'	Q60. Anemia increases risk for pre-eclampsia during pregnancy	radio<br>1 True<br>0 False
82	[q61]<br>Show the field ONLY if:<br>[consent] = '1'	Q61. Anemia can affect the health of both mother and fetus?	radio<br>1 True<br>0 False

83	[q62]<br>Show the field ONLY if:<br>[consent] = '1'	Q62. All pregnant women should have a genotype test done	radio<br>1 True<br>0 False
84	[q63]<br>Show the field ONLY if:<br>[consent] = '1'	Q63. The following are causes of anemia	radio<br>1 Hemoglobinopathies (E.g. Sickle cell disease)<br>2 Menorrhagia (escessive menses)<br>3 Malaria (especially in pregnancy)<br>4 Hook worm infestation
85	[q64]<br>Show the field ONLY if:<br>[consent] = '1'	Q64. Which option is correct regarding common symptoms and signs of anemia:	radio<br>1 Shortness of breath, swelling of the ankles, blurred vision<br>2 Palpitations, shortness of breath, abdominal pain<br>3 Shortness of breath, fatigue, palpitations<br>4 Blurred vision, fatigue, palpitations
86	[q65]<br>Show the field ONLY if:<br>[consent] = '1'	Q65. Which one of the following statements about anemia in pregnancy is correct:	checkbox<br>1 q65___1 All women should take an iron supplement and be counseled on iron rich foods; only women with a history of anemia should be screened for anemia<br>2 q65___2 All women should take an iron supplement, be counseled on iron-rich foods and screened at least once for anemia<br>3 q65___3 All women should be counseled on iron-rich foods and tested for anemia; only women with anemia confirmed by testing should be prescribed or given iron<br>4 q65___4 All women should receive SP for intermittent prevention of malaria<br>5 q65___5 All women should receive medication (eg Mebendazole) for deworming
87	[q66]<br>Show the field ONLY if:<br>[consent] = '1'	Section Header: *Section E: In this section I will ask you a few questions on your knowlegde of obesity*<br>Q66. Is there adult weighing scale in this facility/unit?	radio<br>1 Yes<br>0 No
88	[q67]<br>Show the field ONLY if:<br>[consent] = '1'	Q67. Is the adult weighing scale functional today?	radio<br>1 Yes<br>0 No
89	[q68]<br>Show the field ONLY if:<br>[consent] = '1'	Q68. How long have you been weighing clients?	radio<br>1 Less than 3 years<br>2 3 - 6 years<br>3 7 - 10 years<br>4 11 - 14 years<br>5 More than 15 years
90	[q69]<br>Show the field ONLY if:<br>[consent] = '1'	Q69. Do you have the guidelines/Job Aids/SOPs for wieeght measurement?	radio<br>1 Yes<br>0 No
91	[q70]<br>Show the field ONLY if:<br>[consent] = '1'	Q70. Have you read the guidelines/Job Aids/SOPs for wieght measurement recently?	radio<br>1 Yes<br>0 No

92	[q71]<br>Show the field ONLY if:<br>[consent] = '1'	Q71. When did you last receive training on wieght measurement technique?	radio<br>1 Less than a year<br>2 1 year<br>3 2 years<br>4 3 years<br>5 4 years<br>6 5 years<br>7 6- 10 years<br>8 More than 10 years<br>0 Never
93	[q72]<br>Show the field ONLY if:<br>[consent] = '1'	Q72. Is there a mechanism in place for ensuring that all staff who weigh cients have had appropriate training?	radio<br>1 Yes<br>0 No
94	[q73]<br>Show the field ONLY if:<br>[consent] = '1'	Q73. Do you routinely calibrate adult weighing scale in this facility/unit?<br>*(if no, please skip the next question)*	radio<br>1 Yes<br>0 No<br>95 Don't Know
95	[q74]<br>Show the field ONLY if:<br>[consent] = '1'	Q74. Who is responsible for calibrating the scales in this facility/unit?	notes
96	[q75]<br>Show the field ONLY if:<br>[consent] = '1'	Q75. What is the recommended duration for calibration of weighing scale in this unit?	radio<br>1 6 monthly<br>2 Yearly<br>0 Never<br>97 Other Specify<br>95 Do not know
97	[q75i]<br>Show the field ONLY if:<br>[q75] = '97'	Q75i. Other Specify	notes
98	[q76]<br>Show the field ONLY if:<br>[consent] = '1'	Q76. When was the last time the scale in the unit calibrated? Please specify	notes
99	[q77]<br>Show the field ONLY if:<br>[consent] = '1'	Q77. What class of weighing scale do you use in this unit?	radio<br>1 Class I<br>2 Class II<br>3 Class III<br>95 Do not know
100	[q78]<br>Show the field ONLY if:<br>[consent] = '1'	Q78. Do you routinley measure the height of clients in this unit? (if no, please skip the next question)	radio<br>1 Yes<br>0 No
101	[q79]<br>Show the field ONLY if:<br>[q78] = '1'	Q79. What do you use for height measurement in this unit?	notes
102	[q80]<br>Show the field ONLY if:<br>[consent] = '1'	Q80. What do you understand by "body mass index" (BMI)	radio<br>1 Measurement of weight<br>2 Measurement of height<br>3 Measurement of weight and height<br>95 Do not know

103	[q81]<br>Show the field ONLY if:<br>[consent] = '1'	Q81. Which of the following is true concerning the classification of clients based on BMI (in kg/M2)	radio<br>1 Obesity is BMI> 30-39.9<br>2 Overweight is BMI 25 - 29.9<br>3 Normal weight is BMI 18.5 - 24.9<br>4 Morbidly obese is BMI≥40<br>5 Underweight is BMI < 18.5<br>6 All of the above
104	[q82a]<br>Show the field ONLY if:<br>[consent] = '1'	Section Header: *Section F: Prior Training and Confidence Providing Specific Services 82. Prior Training: ((Please note if you have received training for any of the topics in the table below and the timing of the training))*<br>Q82a. Counseling on healthy diet in an adult	radio (Matrix)<br>0 Never<br>1 Within last 2 years<br>2 Over 2 years ago<br>Field Annotation: Please note if you have received training for any of the topics in the table below and the timing of the training
105	[q82b]<br>Show the field ONLY if:<br>[consent] = '1'	Q82b. Counseling on exercise in an adult	radio (Matrix)<br>0 Never<br>1 Within last 2 years<br>2 Over 2 years ago<br>Field Annotation: Please note if you have received training for any of the topics in the table below and the timing of the training
106	[q82c]<br>Show the field ONLY if:<br>[consent] = '1'	Q82c. Calculating a Body Mass Index (BMI)	radio (Matrix)<br>0 Never<br>1 Within last 2 years<br>2 Over 2 years ago<br>Field Annotation: Please note if you have received training for any of the topics in the table below and the timing of the training
107	[q82d]<br>Show the field ONLY if:<br>[consent] = '1'	Q82d. Normal and abnormal BMI range	radio (Matrix)<br>0 Never<br>1 Within last 2 years<br>2 Over 2 years ago<br>Field Annotation: Please note if you have received training for any of the topics in the table below and the timing of the training
108	[q82e]<br>Show the field ONLY if:<br>[consent] = '1'	Q82e. Diagnosing high blood pressure	radio (Matrix)<br>0 Never<br>1 Within last 2 years<br>2 Over 2 years ago<br>Field Annotation: Please note if you have received training for any of the topics in the table below and the timing of the training
109	[q82f]<br>Show the field ONLY if:<br>[consent] = '1'	Q82f. Classifying high blood pressure in pregnant women (type of hypertensive disorder of pregnancy)	radio (Matrix)<br>0 Never<br>1 Within last 2 years<br>2 Over 2 years ago<br>Field Annotation: Please note if you have received training for any of the topics in the table below and the timing of the training

110	[q82g]<br><br>Show the field ONLY if:<br>[consent] = '1'	Q82g. Treating high blood pressure in pregnant women	radio (Matrix)<br>0 Never<br>1 Within last 2 years<br>2 Over 2 years ago<br><br>Field Annotation: Please note if you have received training for any of the topics in the table below and the timing of the training
111	[q82h]<br><br>Show the field ONLY if:<br>[consent] = '1'	Q82h. Treating high blood pressure in women who are not pregnant	radio (Matrix)<br>0 Never<br>1 Within last 2 years<br>2 Over 2 years ago<br><br>Field Annotation: Please note if you have received training for any of the topics in the table below and the timing of the training
112	[q82i]<br><br>Show the field ONLY if:<br>[consent] = '1'	Q82i. Diagnosing pre-eclampsia in pregnancy	radio (Matrix)<br>0 Never<br>1 Within last 2 years<br>2 Over 2 years ago<br><br>Field Annotation: Please note if you have received training for any of the topics in the table below and the timing of the training
113	[q82j]<br><br>Show the field ONLY if:<br>[consent] = '1'	Q82j. Treating pre-eclampsia in pregnancy	radio (Matrix)<br>0 Never<br>1 Within last 2 years<br>2 Over 2 years ago<br><br>Field Annotation: Please note if you have received training for any of the topics in the table below and the timing of the training
114	[q82k]<br><br>Show the field ONLY if:<br>[consent] = '1'	Q82k. Screening for anemia in pregnancy	radio (Matrix)<br>0 Never<br>1 Within last 2 years<br>2 Over 2 years ago<br><br>Field Annotation: Please note if you have received training for any of the topics in the table below and the timing of the training
115	[q82l]<br><br>Show the field ONLY if:<br>[consent] = '1'	Q82l. Diagnosing anemia in pregnancy	radio (Matrix)<br>0 Never<br>1 Within last 2 years<br>2 Over 2 years ago<br><br>Field Annotation: Please note if you have received training for any of the topics in the table below and the timing of the training
116	[q82m]<br><br>Show the field ONLY if:<br>[consent] = '1'	Q82m. Treating anemia in pregnancy	radio (Matrix)<br>0 Never<br>1 Within last 2 years<br>2 Over 2 years ago<br><br>Field Annotation: Please note if you have received training for any of the topics in the table below and the timing of the training

117	[q82n]<br><br>Show the field ONLY if:<br>[consent] = '1'	Q82n. Screening for diabetes in pregnant women	radio (Matrix)<br>0 Never<br>1 Within last 2 years<br>2 Over 2 years ago<br><br>Field Annotation: Please note if you have received training for any of the topics in the table below and the timing of the training
118	[q82o]<br><br>Show the field ONLY if:<br>[consent] = '1'	Q82o. Diagnosing diabetes in non-pregnant women	radio (Matrix)<br>0 Never<br>1 Within last 2 years<br>2 Over 2 years ago<br><br>Field Annotation: Please note if you have received training for any of the topics in the table below and the timing of the training
119	[q82p]<br><br>Show the field ONLY if:<br>[consent] = '1'	Q82p. Diagnosing diabetes in pregnant women	radio (Matrix)<br>0 Never<br>1 Within last 2 years<br>2 Over 2 years ago<br><br>Field Annotation: Please note if you have received training for any of the topics in the table below and the timing of the training
120	[q82q]<br><br>Show the field ONLY if:<br>[consent] = '1'	Q82q. Treating diabetes in non-pregnant women	radio (Matrix)<br>0 Never<br>1 Within last 2 years<br>2 Over 2 years ago<br><br>Field Annotation: Please note if you have received training for any of the topics in the table below and the timing of the training
121	[q82r]<br><br>Show the field ONLY if:<br>[consent] = '1'	Q82r. Treating diabetes in pregnant women	radio (Matrix)<br>0 Never<br>1 Within last 2 years<br>2 Over 2 years ago<br><br>Field Annotation: Please note if you have received training for any of the topics in the table below and the timing of the training
122	[q83a]<br><br>Show the field ONLY if:<br>[consent] = '1'	Section Header: *Section F Continues: Prior Training and Confidence Providing Specific Services Q83 Confidence: Please note your level of confidence providing care for the topics in the table below*<br>Q83a. Counseling on healthy diet in an adult	radio (Matrix)<br>0 Not confident<br>1 Very confident<br>2 Somewhat confident<br><br>Field Annotation: Please note your level of confidence providing care for the topics in the table below
123	[q83b]<br><br>Show the field ONLY if:<br>[consent] = '1'	Q83b. Counseling on exercise for an adult	radio (Matrix)<br>0 Not confident<br>1 Very confident<br>2 Somewhat confident<br><br>Field Annotation: Please note your level of confidence providing care for the topics in the table below
124	[q83c]<br><br>Show the field ONLY if:<br>[consent] = '1'	Q83c. Calculating a Body Mass Index (BMI)	radio (Matrix)<br>0 Not confident<br>1 Very confident<br>2 Somewhat confident<br><br>Field Annotation: Please note your level of confidence providing care for the topics in the table below

125	[q83d]<br><br>Show the field ONLY if:<br>[consent] = '1'	Q83d. Understanding normal and abnormal BMI range	radio (Matrix)<br>0 Not confident<br>1 Very confident<br>2 Somewhat confident<br><br>Field Annotation: Please note your level of confidence providing care for the topics in the table below
126	[q83e]<br><br>Show the field ONLY if:<br>[consent] = '1'	Q83e. Diagnosing high blood pressure	radio (Matrix)<br>0 Not confident<br>1 Very confident<br>2 Somewhat confident<br><br>Field Annotation: Please note your level of confidence providing care for the topics in the table below
127	[q83f]<br><br>Show the field ONLY if:<br>[consent] = '1'	Q83f. Classifying high blood pressure in pregnant women (type of hypertensive disorder of pregnancy)	radio (Matrix)<br>0 Not confident<br>1 Very confident<br>2 Somewhat confident<br><br>Field Annotation: Please note your level of confidence providing care for the topics in the table below
128	[q83g]<br><br>Show the field ONLY if:<br>[consent] = '1'	Q83g. Treating high blood pressure in pregnant women	radio (Matrix)<br>0 Not confident<br>1 Very confident<br>2 Somewhat confident<br><br>Field Annotation: Please note your level of confidence providing care for the topics in the table below
129	[q83h]<br><br>Show the field ONLY if:<br>[consent] = '1'	Q83h. Treating high blood pressure in women who are not pregnant	radio (Matrix)<br>0 Not confident<br>1 Very confident<br>2 Somewhat confident<br><br>Field Annotation: Please note your level of confidence providing care for the topics in the table below
130	[q83i]<br><br>Show the field ONLY if:<br>[consent] = '1'	Q83i. Diagnosing pre-eclampsia in pregnancy	radio (Matrix)<br>0 Not confident<br>1 Very confident<br>2 Somewhat confident<br><br>Field Annotation: Please note your level of confidence providing care for the topics in the table below
131	[q83j]<br><br>Show the field ONLY if:<br>[consent] = '1'	Q83j. Treating pre-eclampsia in pregnancy	radio (Matrix)<br>0 Not confident<br>1 Very confident<br>2 Somewhat confident<br><br>Field Annotation: Please note your level of confidence providing care for the topics in the table below
132	[q83k]<br><br>Show the field ONLY if:<br>[consent] = '1'	Q83k. Screening for anemia in pregnancy	radio (Matrix)<br>0 Not confident<br>1 Very confident<br>2 Somewhat confident<br><br>Field Annotation: Please note your level of confidence providing care for the topics in the table below

133	[q83l]<br>Show the field ONLY if:<br>[consent] = '1'	Q83l. Diagnosing anemia in pregnancy	radio (Matrix)<br>0 Not confident<br>1 Very confident<br>2 Somewhat confident<br>Field Annotation: Please note your level of confidence providing care for the topics in the table below
134	[q83m]<br>Show the field ONLY if:<br>[consent] = '1'	Q83m. Treating anemia in pregnancy	radio (Matrix)<br>0 Not confident<br>1 Very confident<br>2 Somewhat confident<br>Field Annotation: Please note your level of confidence providing care for the topics in the table below
135	[q83n]<br>Show the field ONLY if:<br>[consent] = '1'	Q83n. Screening for diabetes in pregnant women	radio (Matrix)<br>0 Not confident<br>1 Very confident<br>2 Somewhat confident<br>Field Annotation: Please note your level of confidence providing care for the topics in the table below
136	[q83o]<br>Show the field ONLY if:<br>[consent] = '1'	Q830. Diagnosing diabetes in non-pregnant women	radio (Matrix)<br>0 Not confident<br>1 Very confident<br>2 Somewhat confident<br>Field Annotation: Please note your level of confidence providing care for the topics in the table below
137	[q83p]<br>Show the field ONLY if:<br>[consent] = '1'	Q83p. Diagnosing diabetes in pregnant women	radio (Matrix)<br>0 Not confident<br>1 Very confident<br>2 Somewhat confident<br>Field Annotation: Please note your level of confidence providing care for the topics in the table below
138	[q83q]<br>Show the field ONLY if:<br>[consent] = '1'	Q83q. Treating diabetes in non-pregnant women	radio (Matrix)<br>0 Not confident<br>1 Very confident<br>2 Somewhat confident<br>Field Annotation: Please note your level of confidence providing care for the topics in the table below
139	[q83r]<br>Show the field ONLY if:<br>[consent] = '1'	Q83r. Treating diabetes in pregnant women	radio (Matrix)<br>0 Not confident<br>1 Very confident<br>2 Somewhat confident<br>Field Annotation: Please note your level of confidence providing care for the topics in the table below
140	[antenatal_care]<br>Show the field ONLY if:<br>[consent] = '1'	Section Header: *Usual Practices: Please answer the questions below about your usual practices when you care for women of reproductive age. If you do not provide a specific type of service, please skip to the next service delivery category*<br>Antenatal Care: If you provide ANC services, please answer the questions below. If you do not provide ANC, please skip to the next category of service type	descriptive
141	[anc_provision]<br>Show the field ONLY if:<br>[consent] = '1'	ANC. Do you Provide ANC Services	radio<br>1 Yes<br>0 No

142	[q84]<br>Show the field ONLY if:<br>[anc_provision] = '1'	Q84. Do you check a blood pressure during ANC visits?	radio<br>1 Sometimes<br>2 Most of the time<br>3 Every time
143	[q85]<br>Show the field ONLY if:<br>[anc_provision] = '1'	Q85. Do you check a woman's weight during ANC visits:	radio<br>1 Sometimes<br>2 Most of the time<br>3 Every time
144	[q86]<br>Show the field ONLY if:<br>[anc_provision] = '1'	Q86. Do you discuss a woman's weight with her during ANC visits?	radio<br>1 Sometimes<br>2 Most of the time<br>3 Every time
145	[q87]<br>Show the field ONLY if:<br>[anc_provision] = '1'	Q87. Do you ever calculate a woman's Body Mass Index (BMI) during ANC?	radio<br>1 I am not familiar with "BMI"<br>2 Sometimes<br>3 Most of the time<br>4 Every time
146	[q88]<br>Show the field ONLY if:<br>[anc_provision] = '1'	Q88. Do you talk about healthy diet with women during ANC visits?	radio<br>1 Sometimes<br>2 Most of the time<br>3 Every time
147	[q89]<br>Show the field ONLY if:<br>[anc_provision] = '1'	Q89. Do you talk about exercise during pregnancy?	radio<br>1 Sometimes<br>2 Most of the time<br>3 Every time
148	[q90]<br>Show the field ONLY if:<br>[anc_provision] = '1'	Q90. Do you talk about how much weight a woman should gain during pregnancy?	radio<br>1 Yes<br>0 No
149	[q90a]<br>Show the field ONLY if:<br>[anc_provision] = '1'	Q90a. If yes, do you ever modify the weight you recommend that a woman gain based on her starting weight or Body Mass Index?	radio<br>1 Yes<br>0 No
150	[q91]<br>Show the field ONLY if:<br>[anc_provision] = '1'	Q91. Do you prescribe or give any nutrition supplements for women during pregnancy?	radio<br>1 Yes<br>0 No
151	[q91a]<br>Show the field ONLY if:<br>[q91] = '1'	Q91a. If yes, please check that supplements that you prescribe or give (check all that apply):	radio<br>0 None (just dietary counseling)<br>1 Iron<br>2 Folic acid<br>3 Multivitamin<br>95 Other (please specify)
152	[q91ai]<br>Show the field ONLY if:<br>[q91a] = '95'	Q91ai. Other Specify	notes
153	[q92]<br>Show the field ONLY if:<br>[anc_provision] = '1'	Q92. Do you talk about "pregnancy danger signs" that require urgent evaluation by a health care provider?	radio<br>1 Yes<br>0 No
154	[q93]<br>Show the field ONLY if:<br>[anc_provision] = '1'	Q93. If yes, do you talk about where a woman should go for care if she develops "danger signs"?	radio<br>1 Yes<br>0 No

155	[q94]<br><br>Show the field ONLY if:<br>[anc_provision] = '1'	Q94. Do you check pregnant women for anemia at least once as part of regular ANC?	radio<br>0 No<br>1 Some women<br>2 Most women<br>3 Every women
156	[q95]<br><br>Show the field ONLY if:<br>[q94] = '1' or [q94] = '2' or [q94] = '3'	Q95. If you check pregnant women for anemia, please note which applies in your facility; if you do not check for anemia, please skip to next question	radio<br>1 I refer women to an outside facility for an anemia test?<br>2 I check women for anemia in my facility using a point of care test that provides results at the time of a woman's visit<br>3 I check for anemia in my facility with a laboratory test that requires a woman to follow up for her results
157	[q96]<br><br>Show the field ONLY if:<br>[q94] = '1' or [q94] = '2' or [q94] = '3'	Q96. If you diagnose a woman with anemia during pregnancy; please check all that apply:	radio<br>1 I talk with her about iron-rich foods that she should take<br>2 I prescribe a higher - than normal dose of an iron supplement to treat the anemia<br>3 I do further laboratory tests to understand what type of anemia she has<br>4 I recommend that she see a specialist<br>5 I repeat an anemia test at a later time in pregnancy
158	[q97]<br><br>Show the field ONLY if:<br>[anc_provision] = '1'	Q97. Do you usually check women for diabetes during ANC?	radio<br>1 Yes<br>0 No
159	[q97a]<br><br>Show the field ONLY if:<br>[q97] = '1'	Q97a. If yes, what is your usual practice? Please specify	notes
160	[q98]<br><br>Show the field ONLY if:<br>[anc_provision] = '1'	Q98. Do you provide treatment for pregnant women with diabetes?	radio<br>1 Yes<br>0 No
161	[q99]<br><br>Show the field ONLY if:<br>[q98] = '1'	Q99. If yes, please check all that apply below; otherwise skip to next question	radio<br>1 I discuss a healthy nutrition plan for for diabetes<br>2 I discuss a plan for checking blood sugars<br>3 I prescribe a medication for diabetes if sugars remain high
162	[q99a]<br><br>Show the field ONLY if:<br>[q99] = '3'	Q99a. If yes, check which medications you commonly prescribe:	radio<br>1 Metformin<br>2 Insulin<br>3 Oral sulfonylurea<br>95 Other (please specify)
163	[q99ai]<br><br>Show the field ONLY if:<br>[q99a] = '95'	Q99ai. Other Specify	notes
164	[q100a]<br><br>Show the field ONLY if:<br>[consent] = '1'	Section Header: *"Childbirth: if you provide childbirth services, please answer the questions below. If you do not provide childbirth services, please skip to the next series of questions*<br><br>Q100a. "Do you provide childbirth services?"	radio<br>1 Yes<br>0 No
165	[q100]<br><br>Show the field ONLY if:<br>[q100a] = '1'	Q100. When a woman presents in labor in your facility, which option best characterizes your usual practice:	radio<br>1 I check her blood pressure sometimes<br>2 I check her blood pressure most of the time<br>3 I check her blood pressure every time

166	[q101]<br>Show the field ONLY if:<br>[q100a] = '1'	Q101. During the first 90 minutes after birth, which option best characterizes your usual practice:	radio<br>1 We do not check a woman's blood pressure if it has been normal during labor and childbirth<br>2 We sometimes check her blood pressure<br>3 We almost always check her blood pressure
167	[q102]<br>Show the field ONLY if:<br>[q100a] = '1'	Q102. Before a woman leaves the facility after giving birth, which option best characterizes your practice:	radio<br>1 We do not usually check her blood pressure if it has been normal<br>2 We sometimes check her blood pressure<br>3 We almost always check her blood pressure
168	[q103]<br>Show the field ONLY if:<br>[q100a] = '1'	Q103. If a woman has had high blood pressure during her pregnancy or during labor, birth or the early postnatal period after birth, which option best characterizes your usual practice:	radio<br>1 She should return for a BP check within 2 days<br>2 She should return for a BP check within 2-7 days<br>3 She should return for a BP check within 7-14 days<br>4 She should return for a BP check within 2-4 weeks
169	[q104]<br>Show the field ONLY if:<br>[consent] = '1'	Section Header: *Postnatal care (first 2 months after birth)*<br>Q104. When a woman comes for a postnatal check after being discharged after giving birth, which option best characterizes your practice:	radio<br>1 We do not check her blood pressure if her blood pressure has been normal during pregnancy<br>2 We check her blood pressure sometimes<br>3 We check her blood pressure most of the time
170	[ambulatory_sick_care]<br>Show the field ONLY if:<br>[consent] = '1'	Section Header: *Sick care visits (e.g. cough, fever): If you provide ambulatory sick care, please answer the questions below. If you do not provide sick care visits please skip to the next series of questions.*<br>Do you Provide Ambulatory Sick Care Services.?	radio<br>1 Yes<br>0 No
171	[q105]<br>Show the field ONLY if:<br>[ambulatory_sick_care] = '1'	Q105. Do you check a blood pressure when a woman sees you for an acute illness?	radio<br>1 Sometimes<br>2 Most of the time<br>3 Every time
172	[q106]<br>Show the field ONLY if:<br>[ambulatory_sick_care] = '1'	Q106. Do you check a woman's weight when a woman sees you for an acute illness (e.g. fever, cough)?	radio<br>1 Sometimes<br>2 Most of the time<br>3 Every time
173	[q107]<br>Show the field ONLY if:<br>[consent] = '1'	Section Header: *Family Planning*<br>Q107. Do you check a blood pressure when a woman sees you for Family Planning?	radio<br>1 Sometimes<br>2 Most of the time<br>3 Every time
174	[q108]<br>Show the field ONLY if:<br>[consent] = '1'	Q108. Do you check a woman's weight when a woman sees you for Family Planning?	radio<br>1 Sometimes<br>2 Most of the time<br>3 Every time
175	[q109]<br>Show the field ONLY if:<br>[consent] = '1'	Q109. Do you discuss a woman's weight with her during FP visits?	radio<br>1 Sometimes<br>2 Most of the time<br>3 Every time
176	[latitude_2]<br>Show the field ONLY if:<br>[consent] = '1'	GPS Coordinates: LATITUDE	text<br>Custom alignment: RH<br>Field Annotation: @LATITUDE
177	[longitude_2]<br>Show the field ONLY if:<br>[consent] = '1'	GPS Coordinates: LONGITUDE	text<br>Custom alignment: RH<br>Field Annotation: @LONGITUDE
178	[the_end]<br>Show the field ONLY if:<br>[consent] = '1'	The End. Thank you for participating in this study	descriptive

179	[health_worker_survey_complete]	Section Header: *Form Status*<br>Complete?	dropdown<br>0 Incomplete<br>1 Unverified<br>2 Complete
